# Environmental Risk Factors and Congenital Heart Disease: An Umbrella Review of 165 Systematic Reviews and Meta-Analyses With More Than 120 Million Participants

**DOI:** 10.3389/fcvm.2021.640729

**Published:** 2021-03-11

**Authors:** Tie-Ning Zhang, Qi-Jun Wu, Ya-Shu Liu, Jia-Le Lv, Hui Sun, Qing Chang, Chun-Feng Liu, Yu-Hong Zhao

**Affiliations:** ^1^Department of Clinical Epidemiology, Shengjing Hospital of China Medical University, Shenyang, China; ^2^Clinical Research Center, Shengjing Hospital of China Medical University, Shenyang, China; ^3^Department of Pediatrics, Shengjing Hospital of China Medical University, Shenyang, China

**Keywords:** congenital heart disease, risk factor, environment, epidemiology, meta-analysis

## Abstract

**Background:** The etiology of congenital heart disease (CHD) has been extensively studied in the past decades. Therefore, it is critical to clarify clear hierarchies of evidence between types of environmental factors and CHD.

**Methods:** Electronic searches in PubMed, Embase, Web of Science, Cochrane database were conducted from inception to April 20, 2020 for meta-analyses investigating the aforementioned topic.

**Results:** Overall, 41 studies including a total of 165 meta-analyses of different environmental factors and CHD were examined, covering a wide range of risk factors. The summary random effects estimates were significant at *P* < 0.05 in 63 meta-analyses (38%), and 15 associations (9%) were significant at *P* < 10^−6^. Of these meta-analyses, eventually one risk factor (severe obesity; relative risk: 1.38, 95% confidence interval: 1.30–1.47) had significant summary associations at *P* < 10^−6^, included more than 1,000 cases, had 95% prediction intervals excluding the null value, and were not suggestive of large heterogeneity (*I*^2^ < 50%), small-study effects (*P*-value for Egger's test > 0.10), or excess significance (*P* > 0.10). Eight associations (5%) (including maternal lithium exposure, maternal obesity, maternal alcohol consumption, and maternal fever) had results that were significant at *P* < 10^−6^, included more than 1,000 cases, and had 95% prediction intervals excluding the null value (highly suggestive).

**Conclusion:** This umbrella review shows that many environmental factors have substantial evidence in relation to the risk of developing CHD. More and better-designed studies are needed to establish robust evidence between environmental factors and CHD.

**Systematic Review Registration:** [PROSPERO], identifier [CRD42020193381].

## Introduction

Congenital heart disease (CHD) is defined as a gross structural abnormality of the heart or great vessels (1), and is the most frequently occurring congenital disorder in newborns and the most frequent cause of infant death from birth defects (2, 3). Birth prevalence of CHD is estimated to be 8 cases per 1,000 live births (ranging from 3 to 10) (2), which translates to 1.35 million infants with CHD per year, globally (4). Several studies showed that CHD affects ~2 million families in the United States, which is ~40,000 babies each year in the United States (5–7). Although the etiology of CHD is largely unknown, numerous studies have suggested that the cause of CHD is multifactorial, and both genetic and environmental factors contribute to the development of this disease. In particular, several environmental risk factors, such as maternal factors including obesity and paternal factors including advanced age, are well-accepted major risk factors for CHD in infants. Other reported environmental risk factors mainly include exposure to air pollutants, maternal alcohol consumption, maternal smoking, and maternal exposure to certain drugs during pregnancy (such as antidepressant drugs). However, at present, well-established risk factors for CHD to assist disease prevention are limited.

Numerous meta-analyses and systematic reviews of environmental risk factors associated with CHD have been published. However, to the best of our knowledge, there has been no effort to summarize the evidence from these meta-analyses and systematic reviews, as well as their associated limitations, and thus contribute to better understanding of environmental risk factors for CHD. Therefore, in order to provide an overview of the range and validity of the reported associations of diverse environmental risk factors with CHD in infants, we performed the first umbrella review and summarized the environmental risk factors in previously published meta-analyses and systematic reviews. We assessed the quality and strength of the evidence, evaluated whether there are biases in this evidence and how they manifested, and identified which could be the most robust associations between environmental risk factors and CHD in infants without potential biases.

## Methods

The report of this umbrella review followed the recommendations of the Meta-analysis Of Observational Studies in Epidemiology (MOOSE) guideline (8) and the Preferred Reporting Items for Systematic Reviews and Meta-Analyses (PRISMA) group (9). Before study selection, the protocol for this review was registered with the International Prospective Register of Systematic Reviews (PROSPERO) as CRD42020193381.

### Literature Search and Eligibility Criteria

We conducted computerized literature searches of databases including PubMed, Embase, Web of Science, and the Cochrane database from inception to April 20, 2020 to identify systematic reviews and meta-analyses of epidemiological studies investigating the association between environmental (non-genetic) factors and risk of CHD. The search key words and Medical Subject Heading (MeSH) terms are provided in Supplementary Table 1. We selected potentially relevant articles after title and abstract screening and included eligible articles after full-text review. In addition, the references cited in the retrieved articles were scrutinized by manual search.

Articles were eligible if the authors had performed a systematic search to identify pertinent studies. We included only systematic reviews and meta-analyses of epidemiological studies in humans. We excluded meta-analyses that investigated the association between genetic markers and CHD risk. We also excluded meta-analyses that did not present study specific data [relative risks (RRs), 95% confidence intervals (CIs), and number of cases/controls]. Additionally, studies that examined CHD as a risk factor for other medical conditions or diseases were also excluded. If an article presented separate meta-analyses on more than one eligible exposure factor, these were assessed separately. We did not apply any language restrictions in the selection of eligible studies. When more than one meta-analysis on the same scientific question was eligible, the meta-analysis with the largest number of studies was selected for further analysis, but we conducted sensitivity analyses to assess the concordance of the summary associations (direction, magnitude, and significance) in these duplicate meta-analyses (10).

### Data Extraction

From each eligible meta-analysis, data extraction was done independently by two investigators [T-NZ and Y-SL], and in case of discrepancies, the final decision was that of a third investigator [Q-JW]. We retrieved the first author, year of publication, journal, study design, exposure factors and duration, outcomes, and number of studies. For dose-response meta-analyses, we also retrieved drug dosage exposure factors. If a quantitative synthesis was conducted, we also extracted the study-specific risk estimates [risk ratios, odds ratios (ORs), hazard ratios, or incident risk ratios] together with the corresponding CIs and the number of cases and controls in each study for each risk factor.

### Risk of Bias Assessment

The authors [T-NZ and Y-SL] independently assessed the methodological quality of qualified systematic reviews and meta-analyses using AMSTAR 2 (A Measurement Tool to Assess systematic Reviews) (11). Discrepancies were settled through discussion. The instrument has an overall rating of 16 items related to weaknesses in critical domains (11). In addition, AMSTAR 2 rates the methodological quality of reviews as high, moderate, low, or critically low, instead of creating an overall score (11).

### Statistical Analysis

Estimation of the summary effct—for each unique meta-analysis, we estimated the summary effect and 95% CIs using both fixed and random effects models (12).

Assessment of heterogeneity—heterogeneity between studies was assessed using *I*^2^ statistics. When *I*^2^ exceeds 50 or 75%, heterogeneity is considered large or very large, respectively (13). We also estimated the 95% prediction intervals (95% PIs), which further explained the heterogeneity between studies, and assessed the uncertainty of expected outcomes in new studies dealing with the same association (14).

Evaluation of small-study effects—We used the regression asymmetry test proposed by Egger and colleagues to assess whether there was evidence of a small-study effect (i.e., whether smaller studies tended to give larger effect size estimates than larger studies) (15, 16). A *P* < 0.10 with a more conservative effect in the larger studies is considered evidence of a small-study effect.

Evidence of excess significance bias—We applied the excess statistical significance test, which evaluates whether the observed (O) number of studies with nominally significant results (“positive” studies, *P* < 0.05) is larger than their expected (E) number (17). Two-tailed *P* < 0.10 was considered statistically significant. The expected number of statistically significant studies in each meta-analysis was calculated by summing the statistical power estimates for each study, using an algorithm from a non-central t distribution, and the relative risk estimate of the largest study (i.e., the smallest standard error) was set as the plausible effect size (18). The excess significance test was considered positive when *P* < 0.10 given that O > E.

### Robustness of Evidence

The associations were categorized into strong, highly suggestive, suggestive, or weak according to the following criteria (19): *P* < 10^−6^, >1,000 cases, *P* < 0.05 of the largest study in the meta-analysis, *I*^2^ < 50%, no evidence of small-study effects, no evidence of excess significance bias, the 95% PI excludes the null value for strong evidence; *P* < 10^−6^, >1,000 cases, *P* < 0.05 of the largest study in the meta-analysis for highly suggestive evidence; *P* < 10^−3^, >1,000 cases for suggestive evidence; and *P* < 0.05 for weak evidence. Non-significant associations were those with *P* > 0.05. All analyses were performed using STATA 12.0.

## Results

### Characteristics of the Meta-Analyses

**Figure 1:**
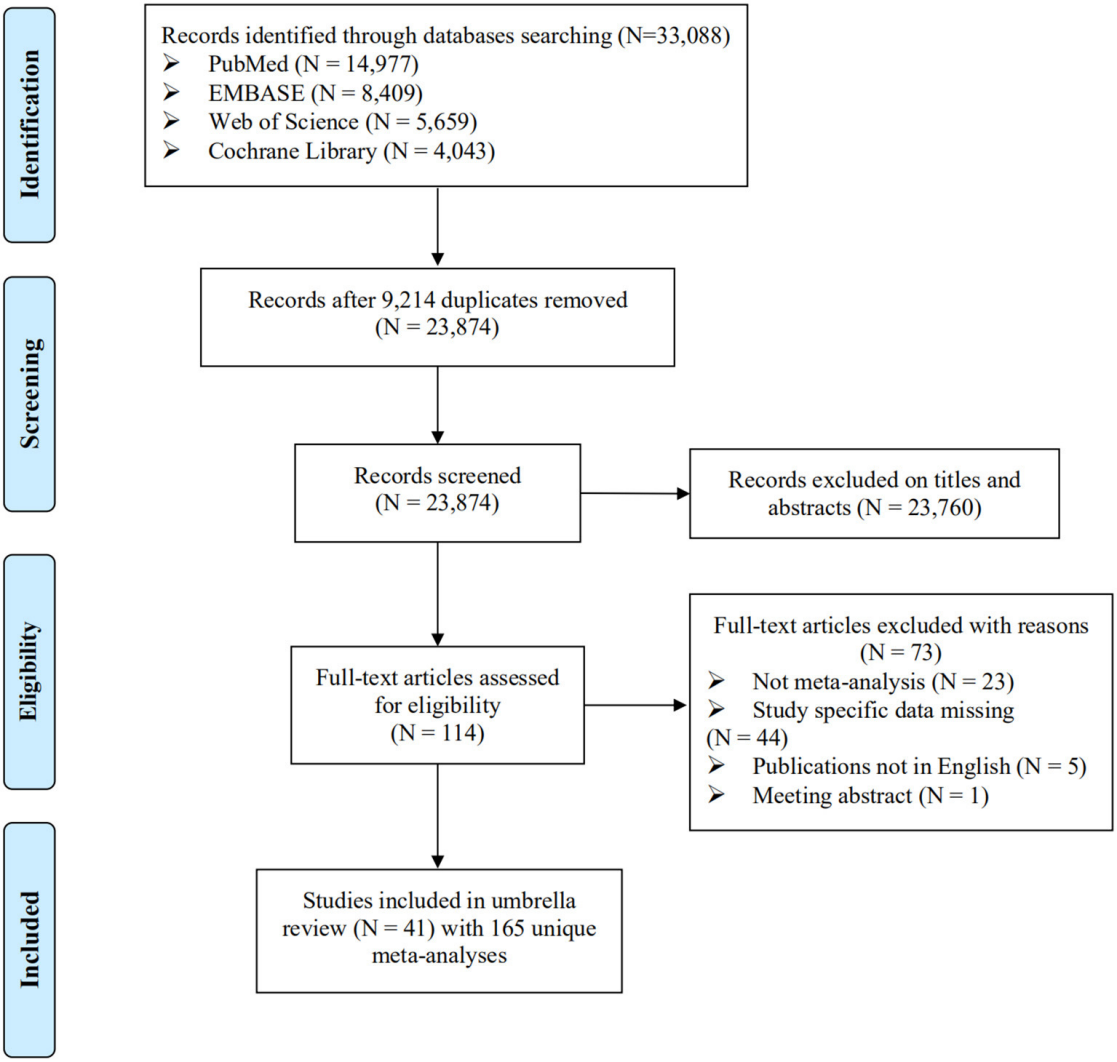
Flowchart of selection of studies for inclusion in umbrella review on environmental factors and CHD.

Overall, 23,874 studies were searched, and 41 studies including a total of 165 meta-analyses were eligible (Figure 1). The characteristics of the 41 studies are shown in Supplementary Table 2. Notably, 73 of 114 articles screened at full text were excluded because of no meta-analyses (*n* = 23), study specific data missing (*n* = 44), not English publications (*n* = 5), and meeting abstract (*n* = 1). The reference list of these excluded studies is shown in Supplementary Table 3.

The included meta-analyses summarized 1,088 individual study estimates. There were three to 55 study estimates combined per meta-analysis, with a median of five studies. The included studies covered 137 associations between different kinds of environmental factors and CHD, over 1.4 million cases and 128 million subjects in total. The median number of cases and total population in each meta-analysis was 2,167 and 589,785, respectively. A total of 101 meta-analyses had at least 1,000 CHD cases.

The 165 meta-analyses covered a wide range of environmental factors. Notably, 54 (33%) of the 165 meta-analyses studied associations between maternal body mass index (BMI) (underweight, overweight, and obesity) and CHD risk (20–22). Twenty-eight (17%) of the 165 meta-analyses explored the association between maternal exposure to different kinds of drugs and CHD risk (23–40). Additionally, other meta-analyses examined associations of exposure to air pollutants (*n* = 16) (41), maternal alcohol consumption (*n* = 13) (42–45), paternal factors (*n* = 13) (46, 47), maternal smoking (*n* = 10) (48–50), maternal metal pollution (*n* = 4) (51, 52), maternal diet factors (*n* = 2) (53, 54), monochorionic twins (*n* = 4) (55), maternal disease (*n* = 6) (56), maternal occupational exposure (*n* = 3) (57), assisted reproductive technology/*in-vitro* fertilization (*n* = 5) (58), maternal reproductive history (*n* = 5) (59), and maternal parity (*n* = 2) (60).

We performed methodological quality assessments of 41 included studies using the AMSTAR 2 questionnaire, which is a revised instrument based on AMSTAR retaining 10 of the original domains and 16 items in total (Supplementary Figure 1). AMSTAR 2 can assist decision makers in the identification of high quality systematic reviews, including those based on non-randomized studies of healthcare interventions. In our study, one included study (32) was considered to be of high quality, two studies (30, 38) were considered of moderate quality, and the remaining studies were assessed as low quality or critically low quality. This was because these 38 studies had one or more critical flaws [usually in item 2 (32/41, 78%) and item 9 (23/41, 56%)] and several non-critical flaws [usually in item 3 (39/41, 95%) and item 10 (40/41, 98%)].

### Summary Effect Size

**Figure 2:**
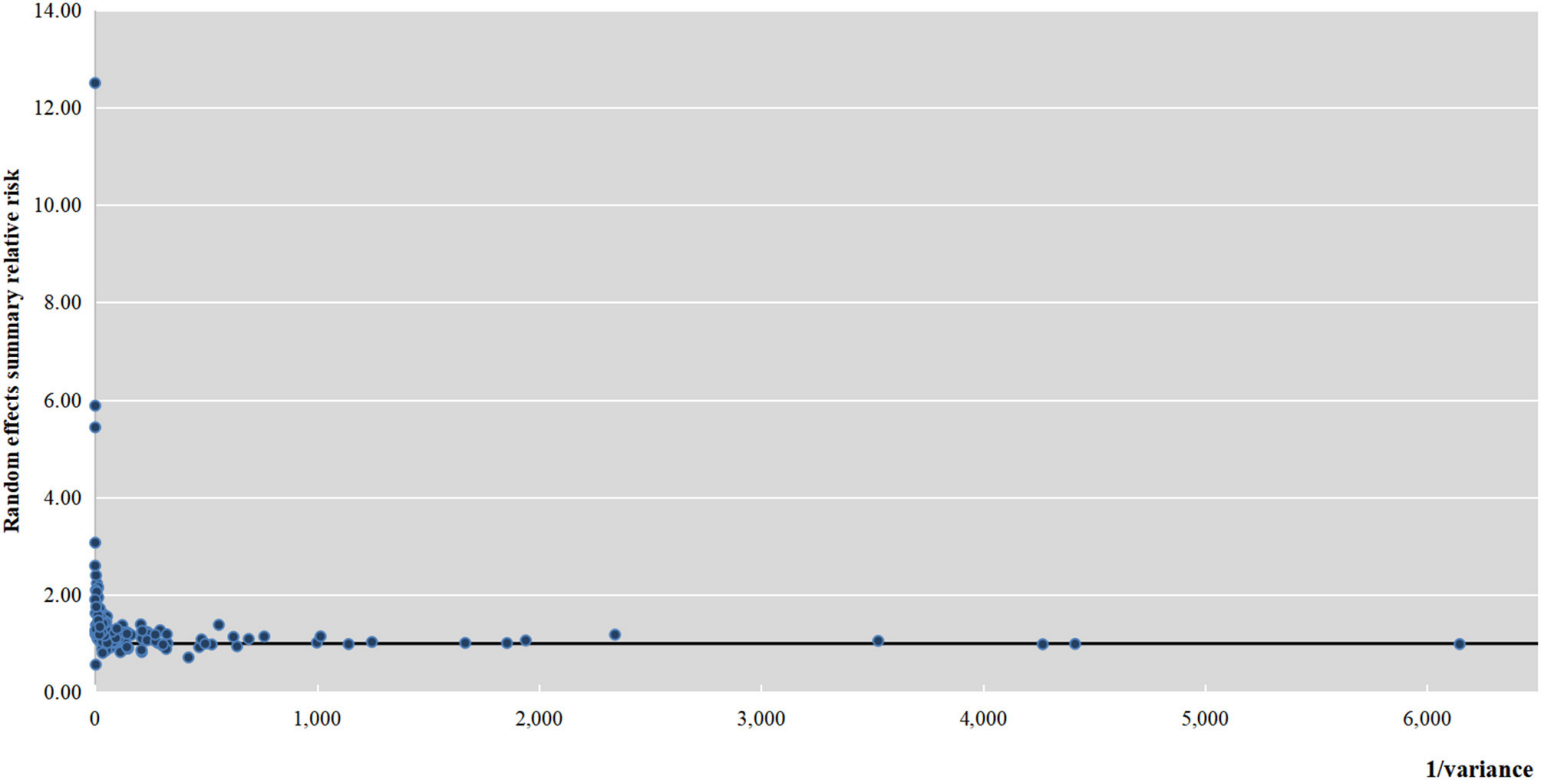
Association of meta-analysis summary effect sizes with inverse of the variance.

The meta-analyses of the 137 associations were re-performed using a fixed-effects and random-effects model. The magnitude of the observed summary random effect estimates ranged from 0.57 to 12.5; 72% of the estimates lay between 1.00 and 2.00 (Figure 2). There were 15 associations that were significant at *P* < 10^−6^ (Supplementary Table 4; Table 1): valproic acid intake, folate intake, lithium exposure (throughout pregnancy/in the first trimester), maternal alcohol consumption, BMI (obesity, moderately obese, and severely obese), obesity as a risk factor for outflow tract defects, obesity as a risk factor for atrial septal defect (ASD), obesity as a risk factor for tetralogy of Fallot (TOF), monochorionic twins (monochorionic twins vs. singletons, monochorionic twins with twin–twin transfusion syndrome (TTTS) vs. singletons, monochorionic twins without TTTS vs. singletons), and maternal fever. Fourteen associations reached *P* < 10^−3^, and the *P*-values of 34 associations were < 0.05. The *P*-values of the remaining 74 associations were not significant. The associations that reached statistical significance (63 in total) indicated that different kinds of environmental factors were related to CHD, including two protective factors (maternal folate supplement and maternal multivitamin supplement) and other 61 risk factors.

**Table 1:** Assessment across the 63 associations of environmental risk factors with CHD.

	**Outcomes**	**Sample size (number of cases)**	**Significance threshold reached (under the random-effects model)**	**95% prediction interval rule**	Estimate of heterogeneity^*^	**Small-study effects or excess significance bias**	**Random-effects summary effect size (95% CI)**
**Associations supported by convincing evidence**							
Severe obesity	CHD	>1,000	<10^−6^	Excluding the null value	Not large	Neither	1.38 (1.30–1.47)
**Associations supported by highly suggestive evidence**							
Lithium (pregnancy)	CHD	>1,000	<10^−6^	Excluding the null value	Not large	Small-study effects/excess significance bias	2.14 (1.67–2.75)
Lithium (in the first trimester)	CHD	>1,000	<10^−6^	Excluding the null value	Not large	Small-study effects/excess significance bias	2.16 (1.69–2.75)
Maternal alcohol consumption	CHD	>1,000	<10^−6^	Excluding the null value	Very large	Small-study effects	1.28 (1.17–1.39)
Obesity	CHD	>1,000	<10^−6^	Excluding the null value	Not large	Excess significance bias	1.18 (1.14–1.22)
Moderate obesity	CHD	>1,000	<10^−6^	Excluding the null value	Not large	Excess significance bias	1.15 (1.09–1.21)
Obesity	Outflow tract defects	>1,000	<10^−6^	Excluding the null value	Not large	Excess significance bias	1.39 (1.26–1.54)
Obesity	ASD	>1,000	<10^−6^	Excluding the null value	Not large	Excess significance bias	1.38 (1.21–1.57)
Maternal fever	CHD	>1,000	<10^−6^	Excluding the null value	Not large	Excess significance bias	1.56 (1.31–1.85)
**Associations supported by suggestive evidence**							
SSRI (in the first trimester)	CHD	>1,000	<10^−3^	Excluding the null value	Large	Neither	1.26 (1.11–1.37)
Maternal folate supplementation	CHD	>1,000	<10^−6^	Including the null value	Very large	Neither	0.72 (0.63–0.89)
Fluoxetine (pregnancy)	CHD	>1,000	<10^−3^	Excluding the null value	Not large	Neither	1.60 (1.32–1.95)
Overweight	Outflow tract defects	>1,000	<10^−3^	Excluding the null value	Not large	Excess significance bias	1.19 (1.09–1.31)
Moderate obesity	ASD	>1,000	<10^−3^	Excluding the null value	Not large	Excess significance bias	1.26 (1.13–1.40)
Severe obesity	ASD	>1,000	<10^−3^	Excluding the null value	Not large	Excess significance bias	1.72 (1.35–2.20)
IVF/intracytoplasmic sperm injection	CHD	>1,000	<10^−3^	Excluding the null value	Not large	Excess significance bias	1.45 (1.20–1.75)
Singleton IVF/intracytoplasmic sperm injection	CHD	>1,000	<10^−3^	Excluding the null value	Not large	Excess significance bias	1.55 (1.21–1.99)
Gravidity number	CHD	>1,000	<10^−3^	Excluding the null value	Not large	Small-study effects/excess significance bias	1.15 (1.08–1.22)
Paternal age 35–39	CHD	>1,000	<10^−3^	Excluding the null value	Not large	Neither	1.14 (1.06–1.22)
Paternal smoking	CHD	>1,000	<10^−3^	Including the null value	Very large	Neither	1.42 (1.17–1.74)
**Associations supported by weak evidence**							
Fluoxetine (in the first trimester)	CHD	>1,000	<0.05	Excluding the null value	Not large	Neither	1.39 (1.12–1.73)
Paroxetine	CHD	>1,000	<0.05	Including the null value	Not large	Neither	1.25 (1.01–1.54)
Nitrofurantoin	HLHS	<1,000	<0.05	Excluding the null value	Not large	Excess significance bias	3.07 (1.59–5.93)
Fluconazole	CHD	>1,000	<0.05	Excluding the null value	Not large	Excess significance bias	1.29 (1.05–1.59)
Valproic acid	CHD	<1,000	<10^−6^	Excluding the null value	Not large	Neither	2.24 (1.65–3.03)
Multivitamin	CHD	>1,000	<0.05	Including the null value	Large	Neither	0.83 (0.70–0.97)
Oral hormone pregnancy tests	CHD	>1,000	<0.05	Excluding the null value	Not large	Neither	1.89 (1.32–2.72)
Air pollution (NO_2_)	COA	<1,000	<0.05	Excluding the null value	Not large	Neither	1.20 (1.02–1.41)
Maternal alcohol consumption	TOF	>1,000	<0.05	Excluding the null value	Not large	Neither	1.19 (1.07–1.33)
Secondhand smoking	CHD	>1,000	<0.05	Excluding the null value	Very large	Small-study effects	2.10 (1.32–3.35)
Smoking	Septal defect	>1,000	<0.05	Excluding the null value	Large	Neither	1.21 (1.01–1.46)
Smoking	Cardiovascular/ heart defects	>1,000	<0.05	Excluding the null value	Large	Neither	1.10 (1.02–1.17)
Smoking	Heart defect	>1,000	<0.05	Excluding the null value	Large	Neither	1.09 (1.00–1.18)
Overweight	CHD	>1,000	<0.05	Including the null value	Large	Neither	1.06 (1.02–1.11)
Overweight	HLHS	<1,000	<0.05	Including the null value	Not large	Small-study effects	1.31 (1.08–1.60)
Moderate obesity	HLHS	<1,000	<10^−3^	Excluding the null value	Not large	Excess significance bias	1.54 (1.21–1.95)
Severe obesity	HLHS	<1,000	<0.05	Including the null value	Not large	Neither	1.60 (1.11–2.31)
Obesity	HLSH	<1,000	<10^−3^	Excluding the null value	Not large	Excess significance bias	1.52 (1.23–1.88)
Severe obesity	TOF	<1,000	<10^−6^	Excluding the null value	Not large	Excess significance bias	1.95 (1.50–2.52)
Obesity	TOF	<1,000	<0.05	Excluding the null value	Not large	Neither	1.27 (1.07–1.51)
Obesity	Conotruncal defects	>1,000	<0.05	Including the null value	Not large	Neither	1.23 (1.08–1.40)
Severe obesity	AVSD	<1,000	<0.05	Including the null value	Not large	Neither	1.44 (1.03–2.00)
Severe obesity	VSD	>1,000	<0.05	Excluding the null value	Not large	Excess significance bias	1.23 (1.07–1.41)
Moderate obesity	COA	<1,000	<0.05	Including the null value	Not large	Neither	1.29 (1.03–1.61)
Obesity	COA	<1,000	<0.05	Including the null value	Not large	Neither	1.25 (1.02–1.53)
Obesity	All septal anomalies	>1,000	<0.05	Excluding the null value	Not large	Small-study effects	1.24 (1.04–1.49)
Chlorination by-products	VSD	<1,000	<0.05	Excluding the null value	Not large	Excess significance bias	1.59 (1.21–2.07)
Monochorionic twins vs. singletons	CHD	<1,000	<10^−6^	Excluding the null value	Not large	Excess significance bias	5.88 (4.18–8.28)
Monochorionic twins with TTTS vs. singletons	CHD	<1,000	<10^−6^	Excluding the null value	Not large	Excess significance bias	12.50 (8.66-18.05)
Monochorionic twins without TTTS vs. singletons	CHD	<1,000	<10^−6^	Excluding the null value	Not large	Excess significance bias	5.44 (3.66–8.08)
Monochorionic twins with TTTS vs. Monochorionic twins without TTTS	CHD	<1,000	<10^−3^	Excluding the null value	Not large	Excess significance bias	2.40 (1.64–3.51)
Maternal fever	VSD	<1,000	<0.05	Including the null value	Not large	Neither	1.34 (1.02–1.78)
Maternal fever	Right obstructive defects	<1,000	<10^−3^	Excluding the null value	Not large	Small-study effects/excess significance bias	2.06 (1.47–2.88)
Occupational exposure to solvents	CHD	>1,000	<0.05	Including the null value	Not large	Neither	1.31 (1.06–1.63)
Gravidity (ever vs. nulligravidity)	CHD	>1,000	<0.05	Excluding the null value	Large	Neither	1.18 (1.03–1.34)
History of spontaneous abortion	CHD	>1,000	<0.05	Including the null value	Not large	Neither	1.18 (1.07–1.31)
History of induced abortion	CHD	>1,000	<0.05	Including the null value	Large	Small-study effects	1.58 (1.12–2.22)
Abortion number	CHD	>1,000	<0.05	Excluding the null value	Large	Excess significance bias	1.31 (1.12–1.52)
Parity number	CHD	>1,000	<0.05	Excluding the null value	Very large	Neither	1.06 (1.02–1.09)
Paternal age ≥40	CHD	>1,000	<0.05	Excluding the null value	Very large	Neither	1.20 (1.05–1.38)
Paternal medium smoking (10–19 cigarettes per day)	CHD	>1,000	<0.05	Excluding the null value	Not large	Neither	1.41 (1.12–1.77)
Paternal heavy smoking (≥20 cigarettes per day)	CHD	>1,000	<0.05	Including the null value	Very large	Small-study effects	1.76 (1.10–2.80)
Paternal wine drinking	CHD	>1,000	<0.05	Including the null value	Very large	Neither	1.48 (1.05–2.07)

* *Heterogeneity was categorized as not large (I^2^ < 50%), large (I^2^ ≥ 50% but I^2^ ≤ 75%), and very large (I^2^ > 75%)*.

*ASD, atrial septal defect; AVSD, atrioventricular septal defect; CHD, congenital heart disease; COA, coarctation of the aorta; HLHS, hypoplastic left heart syndrome; IVF, in-vitro fertilization; SSRI, selective serotonin reuptake inhibitor; TOF, tetralogy of Fallot; TTTS, twin-twin transfusion syndrome; VSD, ventricular septal defect*.

### Heterogeneity

Twenty-four (15%) meta-analyses had high heterogeneity (*I*^2^ ≥ 50%) and 20 (12%) meta-analyses had very high heterogeneity (*I*^2^ > 75%). The meta-analyses with very high heterogeneity examined exposure to a selective serotonin reuptake inhibitor (throughout pregnancy), folate supplement, air pollutants (SO_2_ and CO as risk factors for ventricular septal defect (VSD), and SO_2_ as a risk factor for TOF), maternal alcohol consumption, secondhand smoking, underweight (for outflow tract defects), maternal fever (for left obstructive defects), parity number, paternal age, paternal smoking and alcohol consumption. The 95% PI was also calculated to further assess inter-study heterogeneity. Only the 95% PIs of 14 (8%) meta-analyses excluded the null value (Supplementary Table 4).

### Small-Study Effects

Evidence for small-study effects was noted in 16 (10%) meta-analyses by use of the Egger's test with a more conservative effect in the largest studies. Except for those 16 meta-analyses, there was no evidence for the presence of small-study effects for the other 149 meta-analyses according to Egger's test and comparison between the random effects summary estimate and the point estimate of the largest study (Supplementary Table 4).

### Excess Significance

We detected excess significance using the following criteria: *P* < 0.1 and O > E. As a result, we reported there were 29 meta-analyses in which the excess significance test was positive.

### Robustness of Evidence

Of the 165 eligible meta-analyses, 63 (38%) had nominally significant summary associations (*P* < 0.05) according to a random-effects calculation (Table 1), which showed these meta-analyses presented at least weak evidence. Furthermore, we explored whether the reported associations between different kinds of environmental factors and CHD were supported by convincing, highly suggestive, suggestive, or weak evidence based on different assessment criteria (Table 2). Among all significant associations, only one meta-analysis (2%) was supported by convincing evidence (21). In that study, the authors summarized data on severe obesity during pregnancy and its association with CHD and reported that severe obesity during pregnancy was associated with a higher risk of developing CHD in infants (relative risk 1.38, 95% CI: 1.30–1.47).

**Table 2:** Summary of evidence grading for meta-analyses associating environmental factors and CHD.

	**Criteria used**	**Decreased risk**	**Increased risk**
Convincing (*n* = 1)	Statistical significance at *P* < 10^−6^; >1,000 cases (or >20,000 participants for continuous outcomes); the largest component study reported a significant effect (*P* < 0.05); the 95% prediction interval excluded the null; no large heterogeneity (*I^2^* < 50%); no evidence of small-study effect (*P* > 0.10) and excess significance bias (*P* > 0.10);	None	Severe obesity
Highly suggestive (*n* = 8)	Statistical significance at *P* < 10^−6^; >1,000 cases (or >20,000 participants for continuous outcomes); the largest component study reported a significant effect (*P* < 0.05);	None	**CHD outcome:** Maternal lithium exposure including throughout pregnancy and in the first trimester; Maternal alcohol consumption; Obesity; Moderate obesity; Maternal fever; **Specific subtype of CHD:** Obesity (for ASD and outflow tract defects)
Suggestive (*n* = 11)	Statistical significance at *P* < 10^−3^; >1,000 cases (or >20,000 participants for continuous outcomes);	**CHD outcome:** Maternal folate supplementation	**CHD outcome:** SSRI (in the first trimester); Fluoxetine (pregnancy); IVF/intracytoplasmic sperm injection; Singleton IVF/intracytoplasmic sperm injection; Gravidity number; Paternal age 35–39; Paternal smoking; **Specific subtype of CHD:** Overweight (for outflow tract defects); Moderate obesity (for ASD); Severe obesity (for ASD).
Weak (*n* = 43)	Statistical significance at *P* < 0.05	**CHD outcome:** Maternal multivitamin supplementation	**CHD outcome:** Fluoxetine (in the first trimester); Paroxetine; Fluconazole; Valproic acid; Oral hormone pregnancy tests; Secondhand smoking; Overweight; Monochorionic twins vs. singletons; Monochorionic twins with TTTS vs. singletons; Monochorionic twins without TTTS vs. singletons; Monochorionic twins with TTTS vs. Monochorionic twins without TTTS; Occupational exposure to solvents; Gravidity (ever vs. nulligravidity); History of spontaneous abortion; History of induced abortion; Abortion number; Parity number; Paternal age ≥40; Paternal medium smoking (10–19 cigarettes per day); Paternal heavy smoking (≥20 cigarettes per day); Paternal wine drinking **Specific subtype of CHD:** Nitrofurantoin (for left hypoplastic heart syndrome); Air pollution (NO_2_ for COA); Maternal alcohol consumption (for TOF); Smoking (for septal defect, cardiovascular and/or heart defects); Overweight (for HLHS); Moderate obesity (for HLHS); Severe obesity (for HLHS); Obesity (for HLHS); Severe obesity (for TOF); Obesity (for TOF); Obesity (for conotruncal defects); Severely obese (for AVSD); Severe obesity (for VSD); Moderate obesity (for COA); Obesity (for COA); Obesity (for all septal anomalies); Chlorination by-products (for VSD); Maternal fever (for VSD and right obstructive defects).

*ASD, atrial septal defect; AVSD, atrioventricular septal defect; CHD, congenital heart disease; COA, coarctation of the aorta; HLHS, hypoplastic left heart syndrome; IVF, in-vitro fertilization; SSRI, selective serotonin reuptake inhibitor; TOF, tetralogy of Fallot; TTTS, twin–twin transfusion syndrome; VSD, ventricular septal defect*.

Eight meta-analyses (13%) were supported by highly suggestive evidence (20, 21, 42, 51, 56), and they found positive associations for CHD (with maternal lithium exposure including throughout pregnancy and in the first trimester, maternal alcohol consumption, obesity, moderate obesity, and maternal fever), ASD (with obesity), and outflow tract defects (with obesity). In addition, 11 meta-analyses (17%) were supported by suggestive evidence (21, 31, 33, 38, 46, 58, 59) for an association and the remaining 43 meta-analyses (68%) were supported by weak evidence. Notably, two inverse associations were found for CHD with maternal folate supplementation (suggestive evidence) (31) and maternal multivitamin supplementation (weak evidence) (35).

Finally, we also conducted several sensitivity analyses noting the same associations that would satisfy the same criteria but with fewer individual studies. We noticed that the grading of the evidence for CHD with maternal obesity (highly suggestive evidence), SSRI exposure in the first trimester (suggestive evidence), maternal overweight (weak evidence), and maternal smoking (weak evidence) was not changed. There were no meta-analyses with fewer individual studies that got a higher grading of evidence compared with the meta-analyses that included more individual studies.

## Discussion

### Principal Findings and Possible Explanations

In this umbrella review, we provide an overview and appraisal of environmental risk factors that have been associated with CHD risk and its related subtypes. Overall, our umbrella review examined 63 risk factors that could influence the development of CHD in infants. Notably, one (severe obesity) (21) of these risk factors was supported by evidence with convincing epidemiological credibility, as expressed by large sample size (>1,000 cases), *P* < 10^−6^, low heterogeneity (*I*^2^ < 50%), not suggestive of a small-study effect and excess significant bias, and 95% PIs excluding the null value. In addition, we also reported eight associations (including maternal lithium exposure, maternal obesity, maternal alcohol consumption, and maternal fever) that were supported by evidence with highly suggestive epidemiological credibility. Among these studies with convincing and highly suggestive evidence, the summary effect sizes were relatively large for maternal exposure to lithium (RR > 2).

A possible association between different kinds of environmental factors and the development of CHD has long been speculated, aiming for better prevention and reduced morbidity of CHD from maternal and paternal factors. In our study, 63 of 165 (38%) meta-analyses reported significant results, suggesting their potential role in the process of developing CHD. However, among these nominally significant results, we noticed that 17 (27%) associations had high (or very high) heterogeneity, and 32 (51%) associations had a small-study effect and/or excess significance bias. The applied Egger test may give a spurious signal of small-study effects when there is genuinely high between-study heterogeneity (15). Heterogeneity might often be a manifestation of bias in some studies of a meta-analysis but could also emerge from genuine differences between studies (13, 61). Reasons for heterogeneity include the mixture of cohort studies and case-control studies in some of the meta-analyses, differences in individual studies that included different subtypes of CHDs, differences in assessment of maternal exposure period (such as throughout pregnancy vs. in the first trimester), differences in frequency of exposure in control groups, and differences in the follow-up period and response rates among cases and controls. Therefore, the reported associations with CHD need to be interpreted with caution, in particular for the meta-analyses in which the heterogeneity is high, the number of included studies is relative small, and small-study effects and excess significance bias are evident.

As for CHD, we noticed that maternal severe obesity was a risk factor (RR: 1.38; 95% CI: 1.30–1.47), which was supported by convincing evidence. Similarly, when we analyzed the association between obesity/moderate obesity and CHD, we also found that obesity and moderate obesity could also become risk factors for CHD, which were supported by highly suggestive evidence. Additionally, obesity could become the risk factor for some specific subtypes of CHD. For example, obesity had a tendency to increase the incidence rate of ASD (RR:1.38; 95% CI: 1.21–1.57) and outflow tract defects (RR:1.39; 95% CI: 1.26–1.54), which were also supported by highly suggestive evidence. What's more, the associations between obesity (including moderate obesity and severe obesity) and several subtypes of CHD (including hypoplastic left heart syndrome, TOF, coarctation of the aorta, VSD, atrioventricular septal defect, and conotruncal defects) also passed our evaluation as risk factors with evidence of suggestive or weak epidemiological credibility. All aforementioned associations highly suggested obesity as a risk factor in the process of developing CHD, and several potential mechanisms could explain the associations. Increased fat mass, and in particular increased visceral fat mass, is associated with insulin resistance, hyperinsulinemia, lipo- and glucotoxicity, subclinical inflammation, endothelial dysfunction, and oxidative stress (62, 63). These metabolic, inflammatory, and vascular alterations may generate an adverse impact on the development, gene expression, and function of the placenta, and thus become potentially harmful to the embryo. It has also been proposed that maternal obesity may induce epigenetic changes in the embryo with increased risks of impaired renewal of stem cells and increased risk of malformations (64). Besides, diabetes is more common in overweight and obese individuals, and several studies suggested that diabetes may be a significant risk factor for CHD (65). Unfortunately, we did not perform an evaluation of the association between diabetes and CHD because of specific data missing from the meta-analyses. However, diabetes may have a potential role in CHD and needs to be evaluated in future studies.

Considering the number of studies reporting the association between maternal drugs intake and CHDs, our analyses confirmed the associations. We found that several studies concentrated on the association between maternal exposure to anti-depressive drugs [including serotonin reuptake inhibitor (SRI) and selective SRI (SSRI) such as paroxetine, and fluoxetine] and CHD. We re-performed the different meta-analyses mainly according to the types of anti-depressive drugs as well as the maternal exposure period. Notably, we failed to get any significant association between total SSRI and CHD risk when the maternal exposure period was throughout pregnancy. However, when we focused on the first trimester of pregnancy, we noticed that maternal exposure to total SSRI could increase the risk of CHD (supported by suggestive evidence). This might be because the first trimester of pregnancy is the most critical period for heart development. Additionally, our studies also confirmed that maternal exposure to several specific SSRIs (paroxetine and fluoxetine) could lead to the development of CHD. Considering the high prescription rate of SSRIs in pregnant women with depression and that SSRIs could cross the placenta, especially since previous research has shown that serotonin and serotonin transporters have a significant role in heart development (66), the safety of SSRIs should be discussed with women especially in the first trimester. Besides SSRIs, our studies also suggested that maternal intake of folate (supported by suggestive evidence) and multivitamins (supported by weak evidence) had protective roles in development of CHD. However, of note is the high heterogeneity in these two associations, and thus further studies are still needed to verify the protective roles of folate and multivitamins for CHD.

Quantitative synthesis showed that lithium exposure at any time during pregnancy was associated with a significantly increased risk of CHD (supported by highly suggestive evidence). Notably, we also performed sensitivity analysis to explore the association between lithium exposure and CHD using the study performed by Munk-Olsen et al. but failed to draw any significant results. This discrepancy could be due to the inclusion of a larger sample size in the study performed by Fornaro et al. (*n* = 15,647) (51) than that in the study by Munk-Olsen et al. (*n* = 333) (52). Expanding the sample size and strengthening the statistical power could lead to more convincing results, and we believe there is an association between lithium exposure during pregnancy and CHD in infants.

Maternal alcohol consumption (highly suggestive evidence) and smoking (weak evidence) during pregnancy have also received attention as risk factors for CHD. Recently, the health problems caused by alcohol consumption have become of global public health concern. Although the different individual study results on this association are often inconsistent, we re-evaluated this association and reported maternal alcohol consumption could be a risk factor for CHD through analyses including 55 studies, which contained a large number of cases (*n* = 41,747). An improved understanding of the association between alcohol consumption and CHD may have important public health implications, and could help guide future health education on alcohol-related health risks during pregnancy. In addition, we observed a positive association between maternal smoking during pregnancy and the risk of CHD. The potential mechanism regarding maternal smoking leading to CHD is that *in utero* exposure to nicotine could induce fetal hypoxia and elevate fetal blood pressure (67, 68), and the long-term change in blood pressure can influence the function of cardiac muscles and muscle cells in the aorta (69). Furthermore, a previous study also suggested that genetic regulation was involved in the process of development of CHD, which could be influenced in different ways by the mutagens present in tobacco smoke (70, 71).

Maternal fever in the first trimester appeared to have a positive association with risk of CHD (supported by highly suggestive evidence) (56). Considering this inference was based only on case-control studies, it was necessary to note their potential recall bias and influence in the final conclusion. We know that fever is only a clinical symptom. Among the possible etiologies resulting in fever, infection is the most common pathogenesis mainly including viral, bacterial and fungal organisms. The possible explanation for an association between maternal fever and CHD risk may be attributed to teratogen effects of different kinds of organisms.

Besides the maternal factors, we also reported that paternal factors could influence embryo development and lead to CHD. For example, our study suggested that paternal age, paternal smoking, and even paternal alcohol consumption had impacts on CHD in infants (supported by suggestive and weak evidence). As for paternal age, the mechanism behind such an association is suggested to be that advanced paternal age was previously found to be associated with increased DNA mutations and chromosomal aberrations in sperm (72). Genetic changes in sperm associated with advanced paternal age could lead to an increased risk for birth defects in offspring (73). Similarly, nicotine could affect sperm activity greatly and lead to chromosome aberration, which might affect fetal development, and result in the occurrence of cardiac malformations (46, 74). Besides, paternal smoking could induce maternal passive smoking, and thus may influence embryo development.

### Strengths and Limitations

This umbrella review is the first and the most comprehensive systematic review of meta-analyses on environmental factors and CHD risk. Umbrella reviews have the advantage of building on existing meta-analyses, as opposed to performing new meta-analyses from scratch which would require far more resources with unclear advantages. The robustness and the validity of a total of 63 associations were strictly rated based on the assessment results of a series of statistical analyses. Notably, our umbrella review provided a comprehensive understanding between environmental factors and CHD risk in infants.

Several additional limitations should be considered in the interpretation of our findings. First, some of the caveats pertaining to the interpretation of tests for statistical bias and the potential effect of inflation even in the largest studies, are applicable to all umbrella reviews of risk factors, as previously discussed (13, 61). Second, because of some specific missing data (such as unclear total population numbers of each individual study or unclear total cases in meta-analyses), we excluded some meta-analyses (see Supplementary Table 2), which have explored the association between many other environmental factors and CHD in infants. There may be other environmental factors that could impact on development of CHD in infants, and thus there is a need for future studies. Third, our approach may have missed possible associations that have been published, such as large-scale cohort studies that have not yet been assessed through meta-analyses. This is also the limitation of the methodology of umbrella reviews. Fourth, while we focus on biases such as small-study effects and excess significance bias and other issues including heterogeneity that may have led to false-positive associations, false-negatives are also possible, especially for associations where limited evidence is available. Fifth, AMSTAR-2 found that 38 out of the 41 included meta-analyses studies being of low quality or critically low quality, indicating more studies of high quality are needed in the future. Finally, CHD is a disease that is diagnosed after birth and therefore, some aborted fetuses that may have had CHD would not have been included in the studies. As such, we might have underestimated the influence of each environmental factor on CHD in infants.

## Conclusion

Our umbrella review provides evidence for 63 associations between environmental factors and CHD in infants. Notably, one (severe obesity) of these risk factors for CHD was supported by evidence with convincing epidemiological credibility, eight risk factors for CHD were supported by highly suggestive evidence, and other risk factors for CHD were supported by suggestive or weak evidence. Data from more studies and an investigation of the sources of heterogeneity are needed to examine the associations between other environmental factors and CHD risk in infants. Although the mechanisms of these risk factors are not well-understood, our study provides evidence for researchers and policy makers, and thus generates public health implications for CHD prevention.

## Data Availability Statement

The original contributions presented in the study are included in the article/Supplementary Material, further inquiries can be directed to the corresponding author/s.

## Author Contributions

T-NZ, Q-JW, and Y-HZ conceived the study and contributed to the design. T-NZ, Y-SL, and J-LL conducted the literature search, literature screening, and extracted the data. Q-JW and HS performed the statistical analysis. T-NZ and Q-JW wrote the first draft of the paper. Y-HZ is the guarantor. The corresponding author attests that all listed authors meet authorship criteria and that no others meeting the criteria were omitted. All authors interpreted the data, read the manuscript, and approved the final version.

## Conflict of Interest

The authors declare that the research was conducted in the absence of any commercial or financial relationships that could be construed as a potential conflict of interest.
