# Supplementary material for: Environmental Risk Factors and Congenital Heart Disease: An Umbrella Review of 165 Systematic Reviews and Meta-Analyses With More Than 120 Million Participants
Source: Front Cardiovasc Med. 2021 Mar 11;8:640729. doi: 10.3389/fcvm.2021.640729 (PMC8006458; doi:10.3389/fcvm.2021.640729)
Supplement: Supplementary file 1 [file Data_Sheet_1.PDF]

Supplementary Material

Environmental risk factors and congenital heart disease: an umbrella review of 165 systematic reviews and meta-analyses with more than 120 million participants

Table S1. Search strategy used in the umbrella review

**Literature search strategy in PubMed**
1: malformations OR deformations OR birth defect OR congenital abnormalities OR birth outcome OR obstetrical outcome<br>2: meta-analysis OR systematic review<br>3: 1 AND 2
**Literature search strategy in EMBASE**
1: malformations OR deformations OR birth defect OR congenital abnormalities OR birth outcome OR obstetrical outcome<br>2: ‘meta analysis’ OR ‘systematic review’<br>3: 1 AND 2
**Literature search strategy in Web of Science**
1: Search TS= (malformations OR deformations OR birth defect OR congenital abnormalities OR birth outcome OR obstetrical outcome)<br>2: Search TS= (meta-analysis OR systematic review)<br>3: 1 AND 2
**Literature search strategy in Cochrane library**
1: “malformations” OR “deformations” OR “birth defect” OR “congenital abnormalities” OR “birth outcome” OR “obstetrical outcome”<br>2: “meta-analysis” OR “systematic review”<br>3: 1 AND 2

Table S2. Description of the 41 meta-analyses of environmental factors for CHD.

First author	Year	Journal	Study design included in meta-analysis	Exposure/Factor	Outcome	Type of effect metric
Nikfar et al.(23)	2012	DARU Journal of Pharmaceutical Sciences	Cohort	SSRI	CHD	OR
Rahimi et al.(24)	2006	Reproductive Toxicology	Cohort	SRI	CHD	OR
Painuly et al.(25)	2013	The Psychiatrist	Cohort/Case-control	Paroxetine	CHD	RR
Goldberg et al. (26)	2015	Journal of obstetrics and gynaecology Canada	Cohort/Case-control	Nitrofurantoin	CHD; HLHS	OR; RR
Bracken et al.(27)	1990	Obstetrics & Gynecology	Cohort	Oral contraceptive	CHD	RR
Grigoriadis et al.(28)	2019	Journal of Clinical Psychiatry	Cohort	Benzodiazepine	CHD	OR
Alsaad et al. (29)	2015	Reproductive Toxicology	Cohort	Fluconazole	CHD	OR
Tanoshima et al. (30)	2015	Clinical Pharmacology & Therapeutics	Cohort	Valproic acid	CHD	RR
Feng et al. (31)	2015	Scientific Reports	Cohort/Case-control/RCT	Folate	CHD	RR
De-Regil et al. (32)	2015	Cochrane Database of Systematic Reviews	RCT	Folate	CHD	RR
Riggin et al. (33)	2013	Journal of obstetrics and gynaecology Canada	Cohort	Fluoxetine	CHD	OR
Gao et al. (34)	2017	British Journal of Clinical Pharmacology	Cohort	Fluoxetine	Non-septal defects	RR
Wolf et al. (35)	2017	American Journal of Obstetrics & Gynecology	Cohort/Case-control	Multivitamin	CHD	RR
Zhang et al. (36)	2017	Scientific Reports	Cohort	SSRI	CHD	RR
Selmer et al. (37)	2016	Pharmacoepidemiology and	Cohort	SSRI	CHD	OR

		drug safety				
Gao et al. (38)	2018	BMC Medicine	Cohort	SSRI	CHD	RR
Heneghan et al. (39)	2018	F1000 Research	Cohort/Case-control	Oral hormone pregnancy tests	CHD	OR
Fan et al. (40)	2019	PLoS One	Cohort	Macrolides	CHD; ASD/VSD	OR
Fornaro et al.(51)	2020	American Journal of Psychiatry	Cohort	Lithium	CHD	OR
Munk-Olsen et al. (52)	2018	Lancet Psychiatry	Cohort	Lithium	CHD	OR
Chen et al.(41)	2014	International Journal of Environmental Research and Public Health	Cohort/Case-control	Air pollutants	ASD/VSD/COA/TOF	OR
Zhang et al. (42)	2020	European Journal of Preventive Cardiology	Cohort/Case-control	Maternal alcohol consumption	CHD; ASD/VSD/AVSD/TGA/ TOF/PVS	RR
Wen et al. (43)	2016	Italian Journal of Pediatrics	Cohort/Case-control	Maternal alcohol consumption	CHD	RR
Sun et al. (44)	2015	Congenit Heart Disease	Cohort/Case-control	Maternal alcohol consumption	CHD	RR
Yang et al. (45)	2015	PLoS One	Cohort/Case-control	Maternal alcohol consumption	CHD; VSD	OR
Zheng et al. (48)	2019	Birth	Case-control	Secondhand smoking	CHD	OR
Zhang et al. (49)	2017	The Journal of Maternal-Fetal & Neonatal Medicine	Cohort/Case-control	Smoking	CHD; Conotruncal heart defect; ASD/VSD/AVSD/TGA; Septal defects;	RR
Hackshaw et al. (50)	2011	Human Reproduction	Cohort/Case-control	Smoking	Cardiovascular/heart	OR

		Update			defects	
Zhu et al. (20)	2018	Congenital Heart Disease	Cohort/Case-control	BMI	CHD	RR
Cai et al. (21)	2014	American Journal of Obstetrics & Gynecology	Case-control	BMI	CHD; Hypoplastic left heart syndrome; Outflow tract defects; Conotruncal defects; ASD/TOF/VSD/AVSD/COA/TGA	OR
Stothard et al. (22)	2009	JAMA-The Journal of the American Medical Association	Case-control	BMI	CHD; All septal anomalies; TOF/TGA	OR
Nieuwenhuijsen et al. (53)	2009	Environmental Health Perspectives	Cross-sectional	Chlorination by-products	VSD	OR
Browne et al. (54)	2006	Epidemiology	Cohort/Case-control	Coffee	CHD	OR
Gijtenbeek et al.(55)	2019	Journal of Clinical Medicine	N/A	Monochorionic twins	CHD	RR
Shi et al. (56)	2014	Journal of Perinatology	Case-control	Maternal fever	CHD; ASD/VSD; Right/Left obstructive defects; Conotruncal defects	OR
Spinder et al. (57)	2019	Human Reproduction	Cohort/Case-control	Occupational exposure to solvents/pesticides/metals	CHD	OR
Giorgione et al. (58)	2018	Ultrasound in Obstetrics & Gynecology	Cohort	ART/IVF	CHD; Major/Minor CHD	OR
Feng et al. (59)	2015	Pediatric Cardiology	Case-control	Reproductive history	CHD	OR
Feng et al. (60)	2014	PLoS One	Cohort/Case-control	Parity	CHD	RR
Peng et al. (46)	2019	Clinical Investigation	Case-control	Paternal factors	CHD	OR

Oldereid et al. (47)	2018	Human Reproduction Update	Cohort/Case-control	Paternal factors	CHD	OR

*ART, assisted reproductive technology; ASD, atrial septal defect; AVSD, atrioventricular septal defect; BMI, body mass index; CHD, congenital heart disease; COA, coarctation of the aorta; HLHS, hypoplastic left heart syndrome; IVF, in-vitro fertilization; PVS, pulmonary valve stenosis; SRI, serotonin reuptake inhibitor; SSRI, selective serotonin reuptake inhibitor; TGA, transposition of the great arteries; TOF, tetralogy of Fallot; VSD, ventricular septal defect.

Table S3. References of studies excluded in the umbrella review.

Not meta-analysis (N = 23)

1. Fitton CA, Steiner M, Aucott L, Pell JP, Mackay DF, Fleming M, McLay JS. In utero exposure to antidepressant medication and neonatal and child outcomes: a systematic review. Acta Psychiatr Scand. 2020;141(1):21-33.

2. Abarbanell G, Tepper NK, Farr SL. Safety of contraceptive use among women with congenital heart disease: A systematic review. Congenit Heart Dis. 2019;14(3):331-340.

3. Andrade C. Gestational Exposure to Benzodiazepines, 2: The Risk of Congenital Malformations Examined Through the Prism of Compatibility Intervals. J Clin Psychiatry. 2019;80(5): 19f13081.

4. Lind JN, Interrante JD, Ailes EC, Gilboa SM, Khan S, Frey MT, Dawson AL, Honein MA, Dowling NF, Razzaghi H, Creanga AA, Broussard CS. Maternal Use of Opioids During Pregnancy and Congenital Malformations: A Systematic Review. Pediatrics. 2017;139(6).

5. Carstairs SD. Ondansetron Use in Pregnancy and Birth Defects: A Systematic Review. Obstet Gynecol. 2016;127(5):878-883.

6. Malm H. Prenatal Exposure to Selective Serotonin Reuptake Inhibitors and Infant Outcome. Ther Drug Monit. 2012;34(6):607-614.

7. Wurst KE, Poole C, Ephross SA, Olshan AF. First trimester paroxetine use and the prevalence of congenital, specifically cardiac, defects: a meta-analysis of epidemiological studies. Birth Defects Res A Clin Mol Teratol. 2010;88(3):159-170.

8. Jentink J, Dolk H, Loane MA, Morris JK, Wellesley D, Garne E, de Jong-van DBL. Intrauterine exposure to carbamazepine and specific congenital malformations: systematic review and case-control study. BMJ. 2010;341:c6581.

9. Tuccori M, Montagnani S, Testi A, Ruggiero E, Mantarro S, Scollo C, Pergola A, Fornai M, Antonioli L, Colucci R, Corona T, Blandizzi C. Use of Selective Serotonin Reuptake Inhibitors During Pregnancy and Risk of Major and Cardiovascular Malformations: An Update. Postgrad Med. 2010;122(4):49-65.

10. Tuccori M, Testi A, Antonioli L, Fornai M, Montagnani S, Ghisu N, Colucci R, Corona T, Blandizzi C, Del Tacca M. Safety Concerns Associated With the Use of Serotonin
Reuptake Inhibitors and Other Serotonergic/Noradrenergic Antidepressants During Pregnancy: A Review. Clin Ther. 2009;31(SI1):1426-1453.

11. Lavecchia M, Chari R, Campbell S, Ross S. Ondansetron in Pregnancy and the Risk of Congenital Malformations: A Systematic Review. J Obstet Gynaecol Can. 2018;40(7):910-918.

12. Wilson RD, Davies G, Desilets V, Reid GJ, Summers A, Wyatt P, Young D. The use of folic acid for the prevention of neural tube defects and other congenital anomalies. J Obstet Gynaecol Can. 2003;25(11):959-973.

13. Hendrick V, Suri R, Gitlin MJ, Ortiz-Portillo E. Bupropion Use During Pregnancy: A Systematic Review. Prim Care Companion CNS Disord. 2017;19(5).

14. Nicoll R. Environmental Contaminants and Congenital Heart Defects: A Re-Evaluation of the Evidence. Int J Env Res Pub He. 2018;15(209610).
15. Jacobs M, Zhang G, Chen S, Mullins B, Bell M, Jin L, Guo Y, Huxley R, Pereira G. The association between ambient air pollution and selected adverse pregnancy outcomes in China: A systematic review. Sci Total Environ. 2017;579:1179-1192.
16. Ashworth DC, Elliott P, Toledano MB. Waste incineration and adverse birth and neonatal outcomes: a systematic review. Environ Int. 2014;69:120-132.
17. Irani M, Pakfetrat A, Khadivzadeh T. Maternal exposure to air pollution and fetal abnormalities. Journal of Babol University of Medical Sciences. 2018;20(12):49-59.
18. Parnell AS, Correa A, Reece EA. Pre-pregnancy Obesity as a Modifier of Gestational Diabetes and Birth Defects Associations: A Systematic Review. Matern Child Health J. 2017;21(5):1105-1120.
19. Biagi C, Nunzio MD, Bordoni A, Gori D, Lanari M. Effect of Adherence to Mediterranean Diet during Pregnancy on Children's Health: A Systematic Review. Nutrients. 2019;11(5).
20. Amati F, Hassounah S, Swaka A. The Impact of Mediterranean Dietary Patterns During Pregnancy on Maternal and Offspring Health. Nutrients. 2019;11(10985).
21. Slot A, Eriksen NB, Ringholm L, Damm P, Mathiesen ER. Congenital heart defects in offspring of women with Type 2 diabetes - a systematic review. Dan Med J. 2019;66(6).
22. Warembourg C, Cordier S, Garlantézec R. An update systematic review of fetal death, congenital anomalies, and fertility disorders among health care workers. Am J Ind Med. 2017;60(6):578-590.
23. Baldacci S, Gorini F, Santoro M, Pierini A, Minichilli F, Bianchi F. Environmental and individual exposure and the risk of congenital anomalies: a review of recent epidemiological evidence. Epidemiol Prev. 2018;42(3-4 Suppl 1):1-34.

Study specific data missing (N = 44)

24. Xu A, Cao X, Lu Y, Li H, Zhu Q, Chen X, Jiang H, Li X. A Meta-Analysis of the Relationship Between Maternal Folic Acid Supplementation and the Risk of Congenital Heart Defects. Int Heart J. 2016;57(6):725-728.
25. Wang S, Yang L, Wang L, Gao L, Xu B, Xiong Y. Selective Serotonin Reuptake Inhibitors (SSRIs) and the Risk of Congenital Heart Defects: A Meta-Analysis of Prospective Cohort Studies. J Am Heart Assoc. 2015;4(5).
26. Yakoob MY, Bateman BT, Ho E, Hernandez-Diaz S, Franklin JM, Goodman JE, Hoban RA. The risk of congenital malformations associated with exposure to beta-blockers early in pregnancy: a meta-analysis. Hypertension. 2013;62(2):375-381.
27. Matalon S, Schechtman S, Goldzweig G, Ornoy A. The teratogenic effect of carbamazepine: a meta-analysis of 1255 exposures. Reprod Toxicol. 2002;16(1):9-17.
28. Ingrid GY, Bollano E, Einarson TR, Koren G. Prenatal multivitamin supplementation and rates of congenital anomalies: a meta-analysis. J Obstet Gynaecol Can. 2006;28(8):680-689.

29. O'Brien L, Einarson TR, Sarkar M, Einarson A, Koren G. Does paroxetine cause cardiac malformations? J Obstet Gynaecol Can. 2008;30(8):696-701.
30. Liu D, Zhang C, Wu L, Zhang L, Zhang L. Fetal outcomes after maternal exposure to oral antifungal agents during pregnancy: A systematic review and meta-analysis. Int J Gynaecol Obstet. 2020;148(1):6-13.
31. Kaplan YC, Richardson JL, Keskin-Arslan E, Erol-Coskun H, Kennedy D. Use of ondansetron during pregnancy and the risk of major congenital malformations: A systematic review and meta-analysis. Reprod Toxicol. 2019;86:1-13.
32. Zhang Z, Zhang X, Zhou YY, Jiang CM, Jiang HY. The safety of oral fluconazole during the first trimester of pregnancy: a systematic review and meta-analysis. BJOG. 2019;126(13):1546-1552.
33. Jiang HY, Zhang X, Jiang CM, Fu HB. Maternal and neonatal outcomes after exposure to ADHD medication during pregnancy: A systematic review and meta-analysis. Pharmacoepidemiol Drug Saf. 2019;28(3):288-295.
34. Veroniki AA, Cogo E, Rios P, Straus SE, Finkelstein Y, Kealey R, Reynen E, Soobiah C, Thavorn K, Hutton B, Hemmelgarn BR, Yazdi F, D'Souza J, MacDonald H, Tricco AC. Comparative safety of anti-epileptic drugs during pregnancy: a systematic review and network meta-analysis of congenital malformations and prenatal outcomes. BMC Med. 2017;15(1):95.
35. Shen ZQ, Gao SY, Li SX, Zhang TN, Liu CX, Lv HC, Zhang Y, Gong TT, Xu X, Ji C, Wu QJ, Li D. Sertraline use in the first trimester and risk of congenital anomalies: a systemic review and meta-analysis of cohort studies. Br J Clin Pharmacol. 2017;83(4):909-922.
36. Rough K, Sun JW, Seage GR, Williams PL, Huybrechts KF, Bateman BT, Hernandez-Diaz S. Zidovudine use in pregnancy and congenital malformations. Aids. 2017;31(12):1733-1743.
37. Dellicour S, Sevene E, McGready R, Tinto H, Mosha D, Manyando C, Rulisa S, Desai M, Ouma P, Oneko M, Vala A, Ruperez M, Macete E, Menendez C, Nakanabo-Diallo S,
Kazienga A, Valea I, Calip G, Augusto O, Genton B, Njunju EM, Moore KA, D'Alessandro U, Nosten F, Ter Kuile F, Stergachis A. First-trimester artemisinin derivatives and
quinine treatments and the risk of adverse pregnancy outcomes in Africa and Asia: A meta-analysis of observational studies. Plos Med. 2017;14(5):e1002290.
38. Kang HH, Ahn KH, Hong SC, Kwon BY, Lee EH, Lee JS, Oh MJ, Kim HJ. Association of citalopram with congenital anomalies: A meta-analysis. Obstet Gynecol Sci. 2017;60(2):145-153.
39. Berard A, Iessa N, Chaabane S, Muanda FT, Boukhris T, Zhao JP. The risk of major cardiac malformations associated with paroxetine use during the first trimester of pregnancy: a systematic review and meta-analysis. Br J Clin Pharmacol. 2016;81(4):589-604.
40. Myles N, Newall H, Ward H, Large M. Systematic meta-analysis of individual selective serotonin reuptake inhibitor medications and congenital malformations. Aust N Z

J Psychiatry. 2013; 47(11):1002-1012.
41. Grigoriadis S, VonderPorten EH, Mamisashvili L, Roerecke M, Rehm J, Dennis CL, Koren G, Steiner M, Mousmanis P, Cheung A, Ross LE. Antidepressant exposure during
pregnancy and congenital malformations: is there an association? A systematic review and meta-analysis of the best evidence. J Clin Psychiatry. 2013;74(4):e293-e308.
42. Bar-Oz B, Einarson T, Einarson A, Boskovic R, O'Brien L, Malm H, Berard A, Koren G. Paroxetine and congenital malformations: meta-Analysis and consideration of potential confounding factors. Clin Ther. 2007;29(5):918-926.
43. McKeigue PM, Lamm SH, Linn S, Kutcher JS. Bendectin and birth defects: I. A meta-analysis of the epidemiologic studies. Teratology. 1994;50(1):27-37.
44. Hu CY, Huang K, Fang Y, Yang XJ, Ding K, Jiang W, Hua XG, Huang DY, Jiang ZX, Zhang XJ. Maternal air pollution exposure and congenital heart defects in offspring: A systematic review and meta-analysis. Chemosphere. 2020;253:126668.
45. Zhang TN, Li D, Wu QJ, Xia J, Wen R, Chen XC, Yang N, Chen YL, Huang YH, Liu CF. Exposure to Nitrogen Oxide in the First Trimester and Risk of Cardiovascular-Related Malformations: A Dose-Response Meta-Analysis of Observational Studies. Biomed Res Int. 2018;2018:1948407.
46. Hall KC, Robinson JC. Association between maternal exposure to pollutant particulate matter 2.5 and congenital heart defects: A systematic review. JBI Database of Systematic Reviews and Implementation Reports. 2019;17(8):1695-1716.
47. Vrijheid M, Martinez D, Manzanares S, Dadvand P, Schembari A, Rankin J, Nieuwenhuijsen M. Ambient air pollution and risk of congenital anomalies: a systematic review and meta-analysis. Environ Health Perspect. 2011;119(5):598-606.
48. Lee LJ, Lupo PJ. Maternal smoking during pregnancy and the risk of congenital heart defects in offspring: a systematic review and metaanalysis. Pediatr Cardiol. 2013;34(2):398-407.
49. Zheng Z, Yang T, Chen L, Wang L, Zhang S, Wang T, Zhao L, Ye Z, Chen L, Qin J. Increased maternal Body Mass Index is associated with congenital heart defects: An updated meta-analysis of observational studies. Int J Cardiol. 2018;273:112-120.
50. Liu X, Ding G, Yang W, Feng X, Li Y, Liu H, Zhang Q, Ji L, Li D. Maternal Body Mass Index and Risk of Congenital Heart Defects in Infants: A Dose-Response Meta-Analysis. Biomed Res Int. 2019;2019:1315796.
51. Hwang BF, Jaakkola JJ. Water chlorination and birth defects: a systematic review and meta-analysis. Arch Environ Health. 2003;58(2):83-91.
52. Hoang TT, Marengo LK, Mitchell LE, Canfield MA, Agopian AJ. Original Findings and Updated Meta-Analysis for the Association Between Maternal Diabetes and Risk for Congenital Heart Disease Phenotypes. Am J Epidemiol. 2017;186(1):118-128.
53. Ramakrishnan A, Lee LJ, Mitchell LE, Agopian AJ. Maternal Hypertension During Pregnancy and the Risk of Congenital Heart Defects in Offspring: A Systematic Review and Meta-analysis. Pediatr Cardiol. 2015;36(7):1442-1451.

54. Luteijn JM, Brown MJ, Dolk H. Influenza and congenital anomalies: a systematic review and meta-analysis. Hum Reprod. 2014;29(4):809-823.
55. Lisowski LA, Verheijen PM, Copel JA, Kleinman CS, Wassink S, Visser GH, Meijboom EJ. Congenital heart disease in pregnancies complicated by maternal diabetes mellitus.
An international clinical collaboration, literature review, and meta-analysis. Herz. 2010;35(1):19-26.
56. Xia YQ, Zhao KN, Zhao AD, Zhu JZ, Hong HF, Wang YL, Li SH. Associations of maternal upper respiratory tract infection/influenza during early pregnancy with congenital heart disease in offspring:
evidence from a case-control study and meta-analysis. Bmc Cardiovasc Disor. 2019;19(2771).
57. Simeone RM, Devine OJ, Marcinkevage JA, Gilboa SM, Razzaghi H, Bardenheier BH, Sharma AJ, Honein MA. Diabetes and congenital heart defects: a systematic review, meta-analysis, and modeling project. Am J Prev Med. 2015;48(2):195-204.
58. Dreier JW, Andersen AM, Berg-Beckhoff G. Systematic review and meta-analyses: fever in pregnancy and health impacts in the offspring. Pediatrics. 2014;133(3):e674-e688.
59. Chen L, Yang T, Chen L, Wang L, Wang T, Zhao L, Ye Z, Zhang S, Luo L, Zheng Z, Qin J. Risk of congenital heart defects in offspring exposed to maternal diabetes mellitus: an updated systematic review and meta-analysis. Arch Gynecol Obstet. 2019;300(6):1491-1506.
60. Kalliora C, Mamoulakis C, Vasilopoulos E, Stamatiades GA, Kalafati L, Barouni R, Karakousi T, Abdollahi M, Tsatsakis A. Association of pesticide exposure with human congenital abnormalities. Toxicol Appl Pharm. 2018;346:58-75.
61. Zheng Z, Chen L, Yang T, Yu H, Wang H, Qin J. Multiple pregnancies achieved with IVF/ICSI and risk of specific congenital malformations: a meta-analysis of cohort studies. Reprod Biomed Online. 2018;36(4):472-482.
62. Chen L, Yang T, Zheng Z, Yu H, Wang H, Qin J. Birth prevalence of congenital malformations in singleton pregnancies resulting from in vitro fertilization/intracytoplasmic sperm injection worldwide: a systematic review and meta-analysis. Arch Gynecol Obstet. 2018;297(5):1115-1130.
63. Liang Y, Chen L, Yu H, Wang H, Li Q, Yu R, Qin J. Which type of congenital malformations is significantly increased in singleton pregnancies following after in vitro fertilization/intracytoplasmic sperm injection: a systematic review and meta-analysis. Oncotarget. 2018;9(3):4267-4278.
64. Hoorsan H, Mirmiran P, Chaichian S, Moradi Y, Hoorsan R, Jesmi F. Congenital Malformations in Infants of Mothers Undergoing Assisted Reproductive Technologies: A Systematic Review and Meta-analysis Study. J Prev Med Public Health. 2017;50(6):347-360.
65. Lie RT, Lyngstadaas A, Ørstavik KH, Bakketeig LS, Jacobsen G, Tanbo T. Birth defects in children conceived by ICSI compared with children conceived by other IVF-methods; a meta-analysis. Int J Epidemiol. 2005;34(3):696-701.
66. Yu D, Feng Y, Yang L, Da M, Fan C, Wang S, Mo X. Maternal socioeconomic status and the risk of congenital heart defects in offspring: a meta-analysis of 33 studies.

Plos One. 2014;9(10):e111056.<br>67. Deguen S, Kihal W, Jeanjean M, Padilla C, Zmirou-Navier D. Neighborhood Deprivation and Risk of Congenital Heart Defects, Neural Tube Defects and Orofacial Clefts: A Systematic Review and Meta-Analysis. Plos One. 2016;11(10):e159039.
**Publications not in English (N = 5)**
68. Li SS, Zhang R, Lan X, Qu PF, Dang SN, Chen FY, Yan H. Prenatal exposure to ambient air pollution and congenital heart disease: a Meta-analysis. Zhonghua Liu Xing Bing Xue Za Zhi. 2017;38(8): 1121-1126.<br>69. Pengfei Z, Yi Z, Jie B, Tiantian L, Xiaoming S. Air pollution and adverse birth outcome in China: A comprehensive review. Chinese Journal of Endemiology. 2017;38(3):393-399.<br>70. Baldacci S, Gorini F, Minichilli F, Pierini A, Santoro M, Bianchi F. Review of epidemiological studies on individual and environmental risk factors in the aetiology of congenital heart defects. Epidemiol Prev. 2016;40(3-4):185-196.<br>71. Liu Y, Zhu B, Zhuo L, He MY, Xu Y, Wang TT, Cai QQ, Hu B, Xu JC, Zhang WH. Risk factors for congenital heart disease in Chinese neonates: a Meta analysis. Zhongguo Dang Dai Er Ke Za Zhi. 2017;19(7):754-758.<br>72. Yu ZB, Han SP, Guo XR. A Meta-analysis on the risk factors of perinatal congenital heart disease in Chinese people. Zhonghua Liu Xing Bing Xue Za Zhi. 2008;29(11):1137-1140.
**Meeting abstract (N = 1)**
73. Tzani A, Economopoulos KP. Maternal tobacco use during pregnancy and risk of congenital heart defects in offspring: A systematic review. Tob Induc Dis. 2014;12.

Supplementary Figure 1. The detailed results of methodological quality assessment.

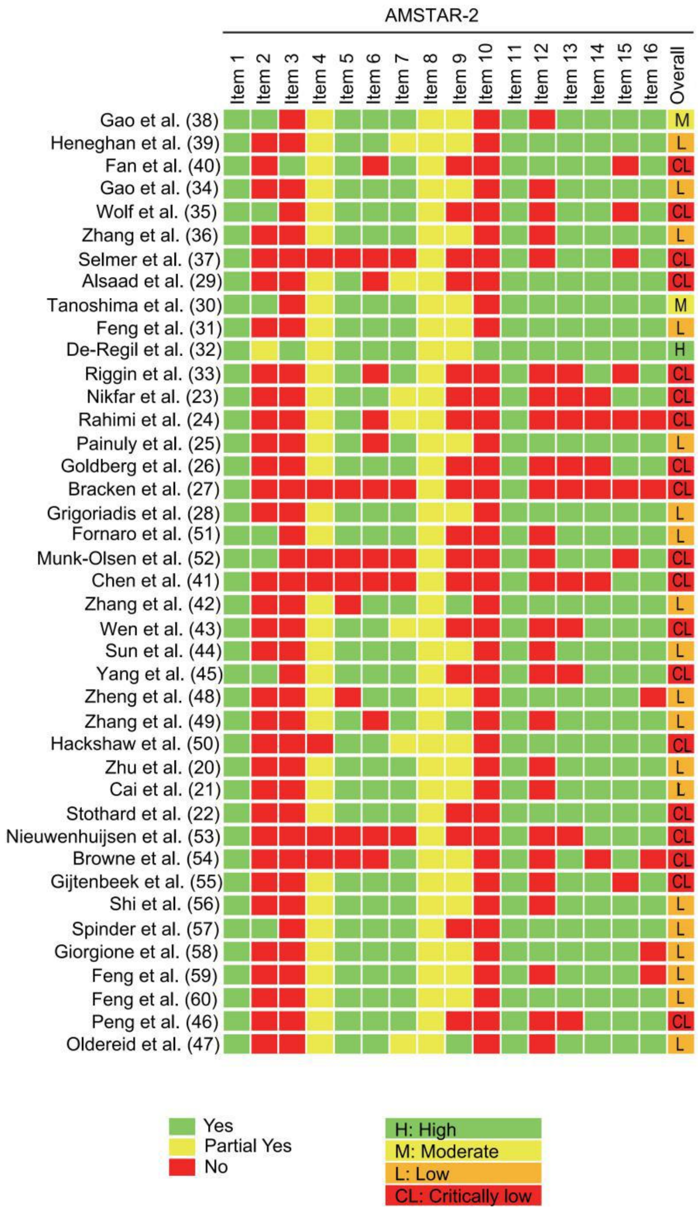

Items
1. Did the research questions and inclusion criteria for the review include the components of PICO?
2. Did the report of the review contain an explicit statement that the review methods were established prior to the conduct of their reivew, and did the report justify any significant deviations for the protocol?
3. Did the review authors explain their selection of the study designs for inclusion in the review?
4. Did the review authors use a comprehensive literature search strategy?
5. Did the review authors perform study selection in duplicate?
6. Did the review authors perform data extraction in duplicate?
7. Did the review authors provide a list of excluded studies and justify the exclusions?
8. Did the review authors describe the included studies in adequate detail?
9. Did the review authors use a satisfactory technique for assessing the risk of bias (RoB) in individual studies that were included in the review?
10. Did the review authors report on the sources of funding for the studies included in the review?
11. If meta-analysis was performed, did the review authors use appropriate methods for statistical combination of the results?
12. If meta-analysis was performed, did the review authors assess the potential impact of RoB in individual studies on the results of the meta-analysis or other evidence synthesis?
13. Did the review authors account for RoB in individual studies when interpreting/discussing the results of the review?
14. Did the review authors provide a satisfactory explanation for, and discussion of, any heterogeneity observed in the results of the review?
15. If they performed quantitative synthesis, did thereview authors carry out an adequate investigation of publications bias (small study bias) and discuss its likely impact on the results of the review?
16. Did the review authors report any potential sources of conflict of interest, including any fundingthey received for conducting the review?

Critical domain: Item 2, 4, 7, 9, 11, 13, 15

Moose Checklist

Item No	Recommendation	Reported on Page No
Reporting of background should include		
1	Problem definition	3
2	Hypothesis statement	3
3	Description of study outcome(s)	3
4	Type of exposure or intervention used	3
5	Type of study designs used	3
6	Study population	3
Reporting of search strategy should include		
7	Qualifications of searchers (eg, librarians and investigators)	3-4
8	Search strategy, including time period included in the synthesis and key words	3-4 Supplementary Table S1
9	Effort to include all available studies, including contact with authors	3-4
10	Databases and registries searched	3-4
11	Search software used, name and version, including special features used (eg, explosion)	3-4
12	Use of hand searching (eg, reference lists of obtained articles)	3-4
13	List of citations located and those excluded, including justification	Supplementary Table S3
14	Method of addressing articles published in languages other than English	3-4
15	Method of handling abstracts and unpublished studies	3-4
16	Description of any contact with authors	None
Reporting of methods should include		
17	Description of relevance or appropriateness of studies assembled for assessing the hypothesis to be tested	3-4
18	Rationale for the selection and coding of data (eg, sound clinical principles or convenience)	None
19	Documentation of how data were classified and coded (eg, multiple raters, blinding and interrater reliability)	None
20	Assessment of confounding (eg, comparability of cases and controls in studies where appropriate)	4-5
21	Assessment of study quality, including blinding of quality assessors, stratification or regression on possible predictors of study results	4-5
22	Assessment of heterogeneity	4-5
23	Description of statistical methods (eg, complete description of fixed or random effects models, justification of whether the chosen models account for predictors of study results, dose-response models, or	4-5

	cumulative meta-analysis) in sufficient detail to be replicated	
24	Provision of appropriate tables and graphics	None
Reporting of results should include		
25	Graphic summarizing individual study estimates and overall estimate	5-6
26	Table giving descriptive information for each study included	Supplementary Table S2
27	Results of sensitivity testing (eg, subgroup analysis)	6-7
28	Indication of statistical uncertainty of findings	None
**Item No**	**Recommendation**	**Reported on Page No**
Reporting of discussion should include		
29	Quantitative assessment of bias (eg, publication bias)	8-9
30	Justification for exclusion (eg, exclusion of non-English language citations)	9
31	Assessment of quality of included studies	8-9
Reporting of conclusions should include		
32	Consideration of alternative explanations for observed results	8-10
33	Generalization of the conclusions (ie, appropriate for the data presented and within the domain of the literature review)	11
34	Guidelines for future research	8-11
35	Disclosure of funding source	12

From: Stroup DF, Berlin JA, Morton SC, et al, for the Meta-analysis Of Observational Studies in Epidemiology (MOOSE) Group. Meta-analysis of Observational Studies in Epidemiology. A Proposal for Reporting. *JAMA*. 2000;283(15):2008-2012. doi: 10.1001/jama.283.15.2008. Transcribed from the original paper within the NEUROSURGERY® Editorial Office, Atlanta, GA, United Sates. August 2012.

PRISMA Checklist

Section/topic	#	Checklist item	Reported on page #
**TITLE**			
Title	1	Identify the report as a systematic review, meta-analysis, or both.	1
**ABSTRACT**			
Structured summary	2	Provide a structured summary including, as applicable: background; objectives; data sources; study eligibility criteria, participants, and interventions; study appraisal and synthesis methods; results; limitations; conclusions and implications of key findings; systematic review registration number.	2
**INTRODUCTION**			
Rationale	3	Describe the rationale for the review in the context of what is already known.	3
Objectives	4	Provide an explicit statement of questions being addressed with reference to participants, interventions, comparisons, outcomes, and study design (PICOS).	3
**METHODS**			
Protocol and registration	5	Indicate if a review protocol exists, if and where it can be accessed (e.g., Web address), and, if available, provide registration information including registration number.	PROSPERO CRD42020193381
Eligibility criteria	6	Specify study characteristics (e.g., PICOS, length of follow-up) and report characteristics (e.g., years considered, language, publication status) used as criteria for eligibility, giving rationale.	3-4
Information sources	7	Describe all information sources (e.g., databases with dates of coverage, contact with study authors to identify additional studies) in the search and date last	3-4

		searched.	
Search	8	Present full electronic search strategy for at least one database, including any limits used, such that it could be repeated.	3-4<br>Table S1
Study selection	9	State the process for selecting studies (i.e., screening, eligibility, included in systematic review, and, if applicable, included in the meta-analysis).	3-4
Data collection process	10	Describe method of data extraction from reports (e.g., piloted forms, independently, in duplicate) and any processes for obtaining and confirming data from investigators.	4
Data items	11	List and define all variables for which data were sought (e.g., PICOS, funding sources) and any assumptions and simplifications made.	4
Risk of bias in individual studies	12	Describe methods used for assessing risk of bias of individual studies (including specification of whether this was done at the study or outcome level), and how this information is to be used in any data synthesis.	4
Summary measures	13	State the principal summary measures (e.g., risk ratio, difference in means).	4-5
Synthesis of results	14	Describe the methods of handling data and combining results of studies, if done, including measures of consistency (e.g., I^2) for each meta-analysis.	4-5
**Section/topic**	**#**	**Checklist item**	**Reported on page #**
Risk of bias across studies	15	Specify any assessment of risk of bias that may affect the cumulative evidence (e.g., publication bias, selective reporting within studies).	5
Additional analyses	16	Describe methods of additional analyses (e.g., sensitivity or subgroup analyses, meta-regression), if done, indicating which were pre-specified.	5

**RESULTS**			
Study selection	17	Give numbers of studies screened, assessed for eligibility, and included in the review, with reasons for exclusions at each stage, ideally with a flow diagram.	3-6<br>Figure 1<br>Table S3
Study characteristics	18	For each study, present characteristics for which data were extracted (e.g., study size, PICOS, follow-up period) and provide the citations.	5-6<br>Table S2
Risk of bias within studies	19	Present data on risk of bias of each study and, if available, any outcome level assessment (see item 12).	5-6
Results of individual studies	20	For all outcomes considered (benefits or harms), present, for each study: (a) simple summary data for each intervention group (b) effect estimates and confidence intervals, ideally with a forest plot.	5-6<br>Table 1
Synthesis of results	21	Present results of each meta-analysis done, including confidence intervals and measures of consistency.	5-6
Risk of bias across studies	22	Present results of any assessment of risk of bias across studies (see Item 15).	5-6
Additional analysis	23	Give results of additional analyses, if done (e.g., sensitivity or subgroup analyses, meta-regression [see Item 16]).	5-6
**DISCUSSION**			
Summary of evidence	24	Summarize the main findings including the strength of evidence for each main outcome; consider their relevance to key groups (e.g., healthcare providers, users, and policy makers).	7-9
Limitations	25	Discuss limitations at study and outcome level (e.g., risk of bias), and at review-level (e.g., incomplete retrieval of identified research, reporting bias).	10-11

Conclusions	26	Provide a general interpretation of the results in the context of other evidence, and implications for future research.	11
**FUNDING**			
Funding	27	Describe sources of funding for the systematic review and other support (e.g., supply of data); role of funders for the systematic review.	12

From: Moher D, Liberati A, Tetzlaff J, Altman DG, The PRISMA Group (2009). Preferred Reporting Items for Systematic Reviews and Meta-Analyses: The PRISMA Statement. PLoS Med 6(7): e1000097. doi:10.1371/journal.pmed1000097

For more information, visit: **www.prisma-statement.org**. Page 2 of 2
