# Supplementary material for: Environmental Risk Factors and Congenital Heart Disease: An Umbrella Review of 165 Systematic Reviews and Meta-Analyses With More Than 120 Million Participants
Source: Front Cardiovasc Med. 2021 Mar 11;8:640729. doi: 10.3389/fcvm.2021.640729 (PMC8006458; doi:10.3389/fcvm.2021.640729)
Supplement: Supplementary file 2 [file Data_Sheet_2.docx]

**Supplementary Table 4. Characteristics, quantitative synthesis, and bias assessment of 41 eligible meta-analyses of environmental factors for CHD.**

|  | **Risk factor** | **Outcomes** | **Number of primary studies** | | **Number of cases** | **Random-effects summary effect size (95% CI)** | **95%PI** | **p random** | **p fixed** | **Largest study**  **Effect size SE**  **(95% CI)** | | **I^2^** | **Egger test p value** |
| --- | --- | --- | --- | --- | --- | --- | --- | --- | --- | --- | --- | --- | --- |
| **Drugs intake during pregnancy** | | | | | | | | | | | | | |
| Nikfar et al. | SSRI (pregnancy) | CHD | 19 | 25,572 | | 1.19 (0.46 - 3.06) | (0.02-69.78) | 0.72 | 3.38*10^-19^ | 1.28 (1.05 - 1.54) | 0.1 | 97.7 | 0.39 |
| Gao et al. | SSRI (in the first trimester) | CHD | 18 | 81,226 | | 1.24 (1.11-1.37) | (0.89-1.71) | 6.25*10^-5^ | 2.36*10^-12^ | 1.15 (1.05 - 1.26) | 0.05 | 59 | 0.45 |
| Rahimi et al. | SRI | CHD | 8 | 21 | | 1.26 (0.44 - 3.58) | (0.34-4.65) | 0.67 | 0.67 | 0.53 (0.05 - 3.71) | 1.11 | 0 | 0.65 |
| Painuly et al. | Paroxetine | CHD | 11 | 23,746 | | 1.25 (1.01 - 1.54) | (0.78-2.01) | 0.04 | 0.01 | 1.03 (0.80 - 1.33) | 0.13 | 30.1 | 0.95 |
| Goldberg et al. | Nitrofurantoin | CHD | 6 | 26,452 | | 0.92 (0.72 - 1.19) | (0.43-1.97) | 0.53 | 0.67 | 1.22 (0.96 - 1.57) | 0.13 | 61 | 0.35 |
| Goldberg et al. | Nitrofurantoin | HLHS | 3 | 305 | | 3.07 (1.59 - 5.93) | (0.04-218.49) | 0.001 | 0.001 | 3.27 (1.59 - 6.71) | 0.37 | 0 | 0.22 |
| Bracken et al. | Oral contraceptive | CHD | 8 | 749 | | 1.38 (0.73 - 2.63) | (0.26-7.28) | 0.32 | 0.24 | 1.25 (0.62 - 2.53) | 0.36 | 49 | 0.47 |
| Grigoriadis et al. | Benzodiazepine | CHD | 4 | 19,321 | | 1.27 (0.98 - 1.65) | (0.72-2.25) | 0.07 | 0.07 | 1.25 (0.92 - 1.70) | 0.16 | 0 | 0.37 |
| Alsaad et al. | Fluconazole | CHD | 3 | 9,617 | | 1.29 (1.05 -1.59) | (0.33-4.98) | 0.02 | 0.02 | 1.28 (1.02 - 1.61) | 0.12 | 0 | 0.28 |
| Tanoshima et al. | Valproic acid | CHD | 20 | 194 | | 2.24 (1.65 - 3.03) | (1.61-3.10) | 2.07*10^-7^ | 2.07*10^-7^ | 1.75 (1.04 - 2.97) | 0.27 | 0 | 0.34 |
| Feng et al. | Folate | CHD | 18 | 18,500 | | 0.72 (0.63- 0.82) | (0.43-1.19) | 9.21*10^-7^ | 1.02*10^-6^ | 1.03 (0.96 - 1.12) | 0.04 | 79.4 | 0 |
| Riggin et al. | Fluoxetine (pregnancy) | CHD | 16 | 35,702 | | 1.60 (1.32 - 1.95) | (1.27-2.02) | 2.49*10^-6^ | 1.78*10^-6^ | 1.56 (1.12 - 2.16) | 0.17 | 1 | 0.68 |
| Gao et al. | Fluoxetine (in the first trimester) | CHD | 9 | 14,240 | | 1.39 (1.12 - 1.73) | (0.98-1.97) | 0.003 | 0.001 | 1.37 (1.05 - 1.79) | 0.14 | 7.3 | 0.84 |
| Wolf et al. | Multivitamin | CHD | 6 | 1,377 | | 0.83 (0.70 - 0.97) | (0.52-1.32) | 0.02 | 0.003 | 0.85 (0.71 - 1.02) | 0.09 | 54.6 | 0.2 |
| Heneghan et al. | Oral hormone pregnancy tests | CHD | 7 | 1,003 | | 1.89 (1.32 - 2.72) | (1.18-3.04) | 0.001 | 0.001 | 2.35 (1.34 - 4.13) | 0.29 | 0 | 0.77 |
| Fan et al. | Macrolides | CHD | 4 | 523 | | 1.14 (0.79 -1.63) | (0.27-4.74) | 0.48 | 0.24 | 0.95 (0.69 - 1.32) | 0.17 | 59.1 | 0.91 |
| Fan et al. | Macrolides | VSD/ASD | 3 | 308 | | 1.16 (0.83 - 1.62) | (0.05-25.06) | 0.39 | 0.4 | 0.91 (0.63 - 1.32) | 0.19 | 31.9 | 0.31 |
| **Metal pollution** | | | | | | | | | | | | | |
| Fornaro et al. | Lithium (pregnancy) | CHD | 9 | 15,647 | | 2.14 (1.67 -2.75) | (1.59-2.89) | 1.88*10^-9^ | 1.88*10^-9^ | 1.86 (1.16 - 2.96) | 0.24 | 0 | 0.06 |
| Fornaro et al. | Lithium (in the first trimester) | CHD | 9 | 15,646 | | 2.16 (1.69 - 2.75) | (1.61-2.89) | 4.69*10^-10^ | 4.69*10^-10^ | 1.96 (1.28 - 3.00) | 0.22 | 0 | 0.08 |
| **Air pollution** | | | | | | | | | | | | | |
| Chen et al. | SO_2_ | VSD | 5 | 3,563 | | 0.99 (0.96-1.02) | (0.90-1.08) | 0.34 | 6.27*10^-6^ | 0.96 (0.95 - 0.97) | 0.01 | 77.9 | 0.62 |
| Chen et al. | PM_10_ | VSD | 5 | 3,629 | | 0.93 (0.84 - 1.02) | (0.69-1.24) | 0.13 | 0.19 | 0.99 (0.94 - 1.04) | 0.03 | 58.7 | 0.05 |
| Chen et al. | NO_2_ | VSD | 5 | 3,874 | | 1.01 (0.90 - 1.12) | (0.77-1.31) | 0.9 | 0.85 | 0.99 (0.84 - 1.15) | 0.08 | 25.9 | 0.6 |
| Chen et al. | CO | VSD | 5 | 3,758 | | 1.11 (0.66 - 1.88) | (0.15-8.29) | 0.7 | 0.11 | 0.99 (0.65 - 1.15) | 0.15 | 91.4 | 0.84 |
| Chen et al. | O_3_ | VSD | 5 | 3,784 | | 0.99 (0.93 - 1.05) | (0.85-1.15) | 0.71 | 0.96 | 1.01 (0.97 - 1.06) | 0.02 | 35.2 | 0.71 |
| Chen et al. | SO_2_ | ASD | 4 | 1,314 | | 0.99 (0.97 - 1.02) | (0.93-1.07) | 0.56 | 0.41 | 0.98 (0.96 - 1.01) | 0.02 | 25.3 | 0.1 |
| Chen et al. | PM_10_ | ASD | 5 | 1,372 | | 1.01 (0.97 - 1.06) | (0.94-1.09) | 0.59 | 0.59 | 1.01 (0.96 - 1.06) | 0.03 | 0 | 0.85 |
| Chen et al. | NO_2_ | ASD | 5 | 1,543 | | 0.96 (0.86 - 1.08) | (0.80-1.16) | 0.5 | 0.5 | 0.93 (0.80 - 1.08) | 0.08 | 0 | 0.22 |
| Chen et al. | CO | ASD | 5 | 1,699 | | 0.97 (0.74 - 1.28) | (0.47-2.01) | 0.82 | 0.75 | 1.33 (1.00 - 1.78) | 0.15 | 34.7 | 0.17 |
| Chen et al. | O_3_ | ASD | 5 | 1,699 | | 1.02 (0.96 - 1.08) | (0.92-1.13) | 0.52 | 0.75 | 1.03 (0.94 - 1.12) | 0.05 | 0 | 0.31 |
| Chen et al. | SO_2_ | COA | 4 | 703 | | 0.99 (0.97 - 1.02) | (0.89-1.10) | 0.41 | 0.32 | 1.01 (0.99 - 1.04) | 0.01 | 67.1 | 0.43 |
| Chen et al. | PM_10_ | COA | 4 | 604 | | 1.06 (0.89 - 1.27) | (0.71-1.58) | 0.51 | 0.51 | 1.10 (0.88 - 1.38) | 0.11 | 0 | 0.52 |
| Chen et al. | NO_2_ | COA | 4 | 645 | | 1.20 (1.02 -1.41) | (0.84-1.70) | 0.03 | 0.03 | 1.24 (1.02 - 1.52) | 0.1 | 0 | 0.39 |
| Chen et al. | SO_2_ | TOF | 4 | 711 | | 0.99 (0.96 - 1.02) | (0.87-1.13) | 0.64 | 0.91 | 1.00 (0.98 - 1.02) | 0.01 | 78.1 | 0.25 |
| Chen et al. | PM_10_ | TOF | 4 | 588 | | 0.85 (0.69 - 1.05) | (0.49-1.47) | 0.12 | 0.1 | 0.91 (0.74 - 1.14) | 0.11 | 7.3 | 0.93 |
| Chen et al. | NO_2_ | TOF | 5 | 620 | | 1.00 (0.78 - 1.29) | (0.58-1.74) | 0.99 | 0.99 | 0.90 (0.59 - 1.38) | 0.22 | 0 | 0.79 |
| **Maternal alcohol consumption** | | | | | | | | | | | | | |
| Zhang et al. | Maternal alcohol consumption | CHD | 55 | 41,747 | | 1.28 (1.17 - 1.39) | (0.70-2.32) | 1.17*10^-7^ | 0.001 | 0.91 (0.85 - 0.99) | 0.04 | 83.1 | 0 |
| Zhang et al. | Maternal alcohol consumption | VSD | 11 | 15,320 | | 1.11 (0.98 - 1.25) | (0.79-1.55) | 0.1 | 0.07 | 0.98 (0.82 - 1.17) | 0.09 | 50.1 | 0.03 |
| Zhang et al. | Maternal alcohol consumption | ASD | 7 | 13,750 | | 1.02 (0.83 - 1.25) | (0.57-1.83) | 0.86 | 0.63 | 0.90 (0.73 - 1.12) | 0.11 | 63 | 0.49 |
| Zhang et al. | Maternal alcohol consumption | AVSD | 3 | 9,638 | | 1.00 (0.73 - 1.37) | (0.13-7.72) | 0.99 | 0.99 | 0.92 (0.58 - 1.48) | 0.24 | 0 | 0.75 |
| Zhang et al. | Maternal alcohol consumption | TGA | 5 | 6,174 | | 1.11 (0.94 - 1.31) | (0.74-1.66) | 0.24 | 0.24 | 1.01 (0.83 - 1.23) | 0.1 | 24.9 | 0.33 |
| Zhang et al. | Maternal alcohol consumption | TOF | 6 | 12,734 | | 1.19 (1.07 - 1.33) | (1.03-1.39) | 0.001 | 0.001 | 1.17 (1.02 - 1.35) | 0.07 | 0 | 0.33 |
| Zhang et al. | Maternal alcohol consumption | PVS | 3 | 12,249 | | 0.87 (0.74 - 1.02) | (0.32-2.38) | 0.08 | 0.08 | 0.82 (0.66 - 1.02) | 0.11 | 0 | 0.34 |
| **Maternal smoking** | | | | | | | | | | | | | |
| Zheng et al. | Secondhand smoking | CHD | 6 | 22,584 | | 2.10 (1.32 - 3.35) | (0.42-10.55) | 0.002 | 6.56*10^-10^ | 1.13 (1.04 - 1.22) | 0.04 | 92.5 | 0.04 |
| Zhang et al. | Smoking | Conotruncal heart defect | 6 | 1,811 | | 1.05 (0.90 - 1.24) | (0.72-1.55) | 0.51 | 0.66 | 1.02 (0.85 - 1.22) | 0.09 | 33.9 | 0.54 |
| Zhang et al. | Smoking | TGA | 3 | 654 | | 1.24 (0.99 -1.54) | (0.30-5.07) | 0.05 | 0.05 | 1.32 (1.02 - 1.71) | 0.13 | 0 | 0.16 |
| Zhang et al. | Smoking | Septal defect | 5 | 2,245 | | 1.21 (1.01 - 1.46) | (0.69-2.11) | 0.04 | 3.61*10^-6^ | 1.40 (1.23 - 1.60) | 0.07 | 57.6 | 0.77 |
| Zhang et al. | Smoking | VSD | 6 | 6,754 | | 0.94 (0.87 - 1.03) | (0.84-1.06) | 0.17 | 0.17 | 0.93 (0.83 - 1.04) | 0.06 | 0.5 | 0.17 |
| Zhang et al. | Smoking | AVSD | 5 | 668 | | 1.31 (0.87 - 1.98) | (0.32-5.37) | 0.19 | 0.01 | 1.60 (1.08 - 2.36) | 0.2 | 71.3 | 0.48 |
| Zhang et al. | Smoking | ASD | 4 | 3,497 | | 1.09 (0.72 - 1.65) | (0.22-5.33) | 0.67 | 0.001 | 1.22 (1.08 - 1.38) | 0.06 | 59.9 | 0.55 |
| Hackshaw et al. | Smoking | Cardiovascular/heart defects | 25 | 29,288 | | 1.10 (1.02 - 1.17) | (0.86-1.40) | 0.01 | 9.21*10^-12^ | 1.15 (1.10 - 1.20) | 0.02 | 63.7 | 0.8 |
| Hackshaw et al. | Smoking | Heart defect | 19 | 26,707 | | 1.09 (1.00 - 1.18) | (0.83-1.43) | 0.05 | 2.05*10^-10^ | 1.15 (1.10-1.20) | 0.02 | 69.3 | 0.87 |
| **Maternal BMI** | | | | | | | | | | | | | |
| Zhu et al. | Underweight | CHD | 17 | 26,165 | | 1.03 (0.98 - 1.09) | (0.90-1.18) | 0.23 | 0.43 | 0.97 (0.91 - 1.04) | 0.03 | 34.9 | 0.28 |
| Zhu et al. | Overweight | CHD | 17 | 32,467 | | 1.06 (1.02 - 1.11) | (0.94-1.21) | 0.004 | 1.22*10^-4^ | 1.02 (0.98 - 1.06) | 0.02 | 60.4 | 0.14 |
| Zhu et al. | Obesity | CHD | 17 | 30,163 | | 1.18 (1.14 - 1.22) | (1.09-1.28) | 1.13*10^-21^ | 6.61*10^-40^ | 1.18 (1.13 - 1.23) | 0.02 | 25.1 | 0.36 |
| Cai et al. | Moderate obesity | CHD | 5 | 18,146 | | 1.15 (1.09 - 1.21) | (1.00-1.32) | 3.93*10^-7^ | 1.63*10^-12^ | 1.20 (1.13 - 1.28) | 0.03 | 33.3 | 0.16 |
| Cai et al. | Severe obesity | CHD | 5 | 16,020 | | 1.38 (1.30 - 1.47) | (1.26-1.53) | 2.09*10^-26^ | 2.09*10^-26^ | 1.38 (1.27 - 1.50) | 0.04 | 0 | 0.1 |
| Cai et al. | Underweight | HLHS | 4 | 379 | | 0.85 (0.60 - 1.19) | (0.40-1.79) | 0.34 | 0.34 | 0.83 (0.83 - 1.48) | 0.29 | 0 | 0.5 |
| Cai et al. | Overweight | HLHS | 4 | 490 | | 1.31 (1.08 - 1.60) | (0.85-2.02) | 0.01 | 0.01 | 1.23 (0.91 - 1.67) | 0.16 | 0 | 0.003 |
| Cai et al. | Moderate obesity | HLHS | 3 | 421 | | 1.54 (1.21 -1.95) | (0.33-7.11) | 3.55*10^-4^ | 3.55*10^-4^ | 1.56 (1.08 - 2.26) | 0.19 | 0 | 0.29 |
| Cai et al. | Severe obesity | HLHS | 3 | 361 | | 1.60 (1.11 - 2.31) | (0.15-17.44) | 0.01 | 0.01 | 1.32 (0.80 - 2.18) | 0.26 | 0 | 0.21 |
| Cai et al. | Obesity | HLHS | 4 | 467 | | 1.52 (1.23 - 1.88) | (0.96-2.41) | 9.20*10^-5^ | 9.20*10^-5^ | 1.47 (1.07 - 2.03) | 0.16 | 0 | 0.17 |
| Cai et al. | Underweight | Outflow tract defects | 3 | 1,645 | | 1.08 (0.68 - 1.72) | (0.01-212.98) | 0.74 | 0.25 | 1.26 (1.04 - 1.52) | 0.1 | 79.8 | 0.98 |
| Cai et al. | Overweight | Outflow tract defects | 3 | 2,344 | | 1.19 (1.09 - 1.31) | (0.66-2.15) | 1.57*10^-4^ | 1.57*10^-4^ | 1.17 (1.04 - 1.32) | 0.06 | 0 | 0.13 |
| Cai et al. | Obesity | Outflow tract defects | 3 | 2,206 | | 1.39 (1.26 - 1.54) | (0.74-2.63) | 2.73*10^-11^ | 2.73*10^-11^ | 1.45 (1.28 - 1.64) | 0.06 | 0 | 0.79 |
| Cai et al. | Underweight | ASD | 5 | 1,983 | | 1.11 (0.85 - 1.45) | (0.50-2.45) | 0.45 | 0.71 | 0.99 (0.82 -1.19) | 0.1 | 56.9 | 0.06 |
| Cai et al. | Overweight | ASD | 4 | 2,382 | | 1.11 (0.94 -1.32) | (0.59-2.11) | 0.22 | 0.18 | 0.97 (0.86 -1.10) | 0.06 | 55.2 | 0.22 |
| Cai et al. | Moderate obesity | ASD | 3 | 2,167 | | 1.26 (1.13 - 1.40) | (0.63-2.52) | 1.90*10^-5^ | 1.90*10^-5^ | 1.22 (1.07 - 1.40) | 0.07 | 0 | 0.45 |
| Cai et al. | Severe obesity | ASD | 3 | 1,880 | | 1.72 (1.35 - 2.20) | (0.15-19.70) | 1.15*10^-5^ | 3.33*10^-9^ | 1.41 (1.07 - 1.85) | 0.14 | 46.5 | 0.39 |
| Cai et al. | Obesity | ASD | 4 | 2,328 | | 1.38 (1.21 -1.57) | (0.92-2.08) | 7.72*10^-7^ | 5.97*10^-10^ | 1.25 (1.10 - 1.42) | 0.07 | 28.3 | 0.27 |
| Cai et al. | Underweight | TOF | 6 | 697 | | 1.01 (0.80 - 1.27) | (0.73-1.39) | 0.94 | 0.94 | 0.98 (0.66 - 1.45) | 0.2 | 0 | 0.85 |
| Cai et al. | Overweight | TOF | 5 | 948 | | 1.15 (0.97 - 1.37) | (0.78-1.69) | 0.11 | 0.04 | 1.30 (1.02 - 1.64) | 0.12 | 17.3 | 0.68 |
| Cai et al. | Moderate obesity | TOF | 3 | 703 | | 1.15 (0.94 - 1.40) | (0.32-4.18) | 0.17 | 0.17 | 1.24 (0.92 - 1.65) | 0.15 | 0 | 0.01 |
| Cai et al. | Severe obesity | TOF | 3 | 648 | | 1.95 (1.50 - 2.52) | (0.36-10.44) | 4.53*10^-7^ | 4.53*10^-7^ | 1.80 (1.27 - 2.55) | 0.18 | 0 | 0.14 |
| Cai et al. | Obesity | TOF | 5 | 887 | | 1.27 (1.07 - 1.51) | (0.91-1.79) | 0.01 | 0.002 | 1.40 (1.09 - 1.81) | 0.13 | 8.7 | 0.48 |
| Cai et al. | Underweight | Conotruncal defects | 3 | 1,078 | | 1.06 (0.88 - 1.29) | (0.31-3.69) | 0.54 | 0.54 | 1.12 (0.87 - 1.43) | 0.13 | 0 | 0.85 |
| Cai et al. | Overweight | Conotruncal defects | 3 | 1,358 | | 1.07 (0.95 - 1.20) | (0.49-2.32) | 0.28 | 0.28 | 1.08 (0.92 - 1.28) | 0.08 | 0 | 0.02 |
| Cai et al. | Obesity | Conotruncal defects | 3 | 1,278 | | 1.23 (1.08 - 1.40) | (0.52-2.89) | 0.002 | 0.002 | 1.19 (0.99 - 1.43) | 0.09 | 0 | 0.13 |
| Cai et al. | Underweight | AVSD | 3 | 807 | | 0.83 (0.66 - 1.03) | (0.20-3.44) | 0.09 | 0.09 | 0.84 (0.67 - 1.07) | 0.12 | 0 | 0.21 |
| Cai et al. | Overweight | AVSD | 3 | 862 | | 0.90 (0.75 - 1.07) | (0.28-2.86) | 0.23 | 0.23 | 0.93 (0.76 - 1.14) | 0.1 | 0 | 0.58 |
| Cai et al. | Moderate obesity | AVSD | 3 | 824 | | 1.04 (0.85 - 1.28) | (0.28-3.92) | 0.7 | 0.7 | 1.10 (0.88 - 1.38) | 0.12 | 0 | 0.16 |
| Cai et al. | Severe obesity | AVSD | 3 | 756 | | 1.44 (1.03 - 2.00) | (0.17-12.40) | 0.03 | 0.03 | 1.37 (0.92 - 2.04) | 0.2 | 0 | 0.37 |
| Cai et al. | Obesity | AVSD | 3 | 861 | | 1.11 (0.93 - 1.33) | (0.34-3.59) | 0.24 | 0.24 | 1.15 (0.94 - 1.41) | 0.1 | 0 | 0.17 |
| Cai et al. | Underweight | VSD | 5 | 4,936 | | 0.98 (0.90- 1.07) | (0.85-1.13) | 0.69 | 0.69 | 0.98 (0.86 - 1.12) | 0.07 | 0 | 0.26 |
| Cai et al. | Overweight | VSD | 4 | 5,509 | | 0.99 (0.91 - 1.09) | (0.75-1.33) | 0.97 | 0.69 | 0.96 (0.88 - 1.05) | 0.05 | 29.2 | 0.21 |
| Cai et al. | Moderate obesity | VSD | 3 | 4,972 | | 1.01 (0.90 - 1.14) | (0.33-3.16) | 0.81 | 0.71 | 0.97 (0.87 - 1.07) | 0.05 | 45.4 | 0.88 |
| Cai et al. | Severe obesity | VSD | 3 | 4,409 | | 1.23 (1.07 - 1.41) | (0.50-3.03) | 0.004 | 0.004 | 1.23 (1.00 - 1.51) | 0.11 | 0 | 0.71 |
| Cai et al. | Obesity | VSD | 4 | 5,230 | | 1.05 (0.94 - 1.17) | (0.70-1.56) | 0.4 | 0.21 | 1.01 (0.91 - 1.11) | 0.05 | 44 | 0.56 |
| Cai et al. | Underweight | COA | 4 | 449 | | 0.87 (0.64 - 1.19) | (0.44-1.71) | 0.38 | 0.38 | 0.96 (0.64 - 1.45) | 0.21 | 0 | 0.56 |
| Cai et al. | Overweight | COA | 4 | 575 | | 1.21 (0.95 - 1.54) | (0.55-2.65) | 0.12 | 0.08 | 1.07 (0.83 - 1.39) | 0.13 | 30.2 | 0.01 |
| Cai et al. | Moderate obesity | COA | 3 | 502 | | 1.29 (1.03 - 1.61) | (0.31-5.37) | 0.02 | 0.02 | 1.29 (0.96 - 1.73) | 0.15 | 0 | 0.63 |
| Cai et al. | Severe obesity | COA | 3 | 424 | | 1.10 (0.70 - 1.71) | (0.03-36.97) | 0.69 | 0.71 | 0.78 (0.42 - 1.45) | 0.32 | 15 | 0.42 |
| Cai et al. | Obesity | COA | 4 | 534 | | 1.25 (1.02 - 1.53) | (0.80-1.94) | 0.03 | 0.03 | 1.28 (0.97 - 1.69) | 0.14 | 0 | 0.36 |
| Cai et al. | Underweight | TGA | 6 | 653 | | 0.90 (0.62 - 1.31) | (0.32-2.54) | 0.59 | 0.75 | 1.32 (0.90 - 1.94) | 0.2 | 50 | 0.53 |
| Cai et al. | Overweight | TGA | 5 | 724 | | 0.93 (0.78 - 1.10) | (0.70-1.23) | 0.38 | 0.38 | 0.88 (0.66 - 1.18) | 0.15 | 0 | 0.27 |
| Cai et al. | Moderate obesity | TGA | 3 | 543 | | 1.04 (0.76 - 1.44) | (0.04-26.34) | 0.8 | 0.81 | 1.09 (0.76 - 1.58) | 0.19 | 46.8 | 0.58 |
| Cai et al. | Severe obesity | TGA | 3 | 485 | | 1.19 (0.63 - 2.25) | (0.00-1304.88) | 0.59 | 0.59 | 0.70 (0.40 - 1.25) | 0.29 | 63.3 | 0.003 |
| Cai et al. | Obesity | TGA | 5 | 678 | | 1.01 (0.78 - 1.31) | (0.51-2.00) | 0.92 | 0.96 | 0.95 (0.69 - 1.32) | 0.16 | 34.2 | 0.82 |
| Stothard et al. | Obesity | All septal anomalies | 4 | 3,483 | | 1.24 (1.04 - 1.49) | (0.72-2.14) | 0.02 | 9.80*10^-5^ | 1.18 (1.08 - 1.30) | 0.05 | 9.9 | 0.09 |
| **Maternal diet** | | | | | | | | | | | | | |
| Nieuwenhuijsen et al. | Chlorination by-products | VSD | 3 | 746 | | 1.59 (1.21 - 2.07) | (0.28-8.99) | 0.001 | 0.001 | 1.43 (1.00- 2.04) | 0.18 | 0 | 0.14 |
| Browne et al. | Coffee | CHD | 4 | 4,068 | | 1.16 (0.90 - 1.50) | (0.45-2.98) | 0.24 | 0.03 | 1.30 (1.00 - 1.60) | 0.12 | 47.9 | 0.34 |
| **Monochorionic twins** | | | | | | | | | | | | | |
| Gijtenbeek et al. | Monochorionic twins vs.singletons | CHD | 6 | 141 | | 5.88 (4.18 - 8.28) | (3.62-9.55) | 3.58*10^-24^ | 3.58*10^-24^ | 8.28 (4.16 - 16.49) | 0.35 | 0 | 0.03 |
| Gijtenbeek et al. | Monochorionic twins with TTTS vs.singletons | CHD | 6 | 58 | | 12.50 (8.66 - 18.05) | (7.43-21.04) | 2.08*10^-41^ | 2.08*10^-41^ | 18.74 (8.36 - 42.00) | 0.41 | 0 | 0.04 |
| Gijtenbeek et al. | Monochorionic twins without TTTS vs.singletons | CHD | 5 | 99 | | 5.44 (3.66 - 8.08) | (2.86-10.35) | 5.58*10^-17^ | 5.58*10^-17^ | 7.25 (3.60 - 14.58) | 0.36 | 0 | 0.07 |
| Gijtenbeek et al. | Monochorionic twins with TTTS vs. Monochorionic twins without TTTS | CHD | 4 | 123 | | 2.40 (1.64 - 3.51) | (1.04-5.54) | 6.65*10^-6^ | 6.65*10^-6^ | 2.59 (1.50 - 4.44) | 0.28 | 0 | 0.67 |
| **Maternal disease** | | | | | | | | | | | | | |
| Shi et al. | Maternal fever | CHD | 7 | 1,536 | | 1.56 (1.31 - 1.85) | (1.03-2.34) | 3.61*10^-7^ | 1.62*10^-12^ | 1.69 (1.37 - 2.08) | 0.11 | 37.2 | 0.85 |
| Shi et al. | Maternal fever | VSD | 4 | 490 | | 1.34 (1.02 - 1.78) | (0.62-2.91) | 0.04 | 0.03 | 1.07 (0.88 - 1.87) | 0.19 | 14 | 0.88 |
| Shi et al. | Maternal fever | Right obstructive defects | 3 | 411 | | 2.06 (1.47 - 2.88) | (0.23-18.07) | 2.36*10^-5^ | 2.36*10^-5^ | 1.88 (1.18 - 2.99) | 0.24 | 0 | 0.1 |
| Shi et al. | Maternal fever | ASD | 4 | 449 | | 1.62 (0.80 - 3.31) | (0.09-28.56) | 0.18 | 0.02 | 2.62 (1.28 - 5.37) | 0.37 | 60 | 0.7 |
| Shi et al. | Maternal fever | Conotruncal defects | 5 | 567 | | 1.23 (0.92 - 1.66) | (0.76-1.99) | 0.16 | 0.16 | 0.96 (0.57 - 1.60) | 0.26 | 0 | 0.24 |
| Shi et al. | Maternal fever | Left obstructive defects | 3 | 396 | | 1.26 (0.45 - 3.49) | (0.00-250567.61) | 0.66 | 0.03 | 2.78 (1.62 - 4.76) | 0.28 | 81.2 | 0.47 |
| **Maternal occupational exposure** | | | | | | | | | | | | | |
| Spinder et al. | Occupational exposure to solvents | CHD | 6 | 2,526 | | 1.31 (1.06 - 1.63) | (0.97-1.78) | 0.01 | 0.01 | 1.20 (0.90 - 1.60) | 0.15 | 0 | 0.54 |
| Spinder et al. | Occupational exposure to pesticides | CHD | 5 | 4,742 | | 0.81 (0.54 - 1.21) | (0.27-2.44) | 0.3 | 0.74 | 1.04 (0.93 - 1.15) | 0.05 | 38.4 | 0.02 |
| Spinder et al. | Occupational exposure to metals | CHD | 3 | 1,185 | | 1.90 (0.67 - 5.38) | (0.00-54876.16) | 0.23 | 0.09 | 0.94 (0.23 - 2.75) | 0.63 | 44.2 | 0.87 |
| **IVF/ART** | | | | | | | | | | | | | |
| Giorgione et al. | IVF/intracytoplasmic sperm injection | CHD | 8 | 2,289 | | 1.45 (1.20 - 1.75) | (0.89-2.35) | 1.10*10^-4^ | 5.63*10^-8^ | 1.53 (1.19 - 1.97) | 0.13 | 43.5 | 0.61 |
| Giorgione et al. | IVF/intracytoplasmic sperm injection | major congenital heart defects（included TOF, TGA, HLHS, COA and AVSD） | 3 | 44 | | 0.57 (0.14 - 2.30) | (0.00-637133.63) | 0.43 | 0.79 | 1.14 (0.55 - 2.34) | 0.37 | 39 | 0.23 |
| Giorgione et al. | IVF/intracytoplasmic sperm injection | Minor congenital heart defects （included VSD and ASD） | 3 | 142 | | 1.30 (0.66 - 2.54) | (0.00-1532.51) | 0.45 | 0.14 | 1.32 (0.90 - 1.95) | 0.2 | 53.8 | 0.96 |
| Giorgione et al. | singleton IVF/intracytoplasmic sperm injection | CHD | 5 | 1,854 | | 1.55 (1.21 - 1.99) | (0.80-3.01) | 4.97*10^-4^ | 3.20*10^-5^ | 1.39 (1.01 - 1.92) | 0.16 | 34.8 | 0.11 |
| **Maternal reproductive history** | | | | | | | | | | | | | |
| Feng et al. | Gravidity (ever vs. nulligravidity) | CHD | 10 | 5,464 | | 1.18 (1.03 - 1.34) | (0.80-1.72) | 0.01 | 1.03*10^-5^ | 1.22 (1.09 - 1.36) | 0.06 | 61.9 | 0.63 |
| Feng et al. | Gravidity number | CHD | 7 | 4,381 | | 1.15 (1.08 - 1.22) | (0.98-1.34) | 1.47*10^-5^ | 2.89*10^-8^ | 1.08 (1.01 - 1.16) | 0.04 | 42.5 | 0.09 |
| Feng et al. | History of spontaneous abortion | CHD | 11 | 7,240 | | 1.18 (1.07 - 1.31) | (0.95-1.48) | 0.001 | 1.28*10^-5^ | 1.13 (0.98 - 1.31) | 0.07 | 28.2 | 0.77 |
| Feng et al. | History of induced abortion | CHD | 6 | 2,499 | | 1.58 (1.12 - 2.22) | (0.58-4.35) | 0.01 | 0.001 | 1.22 (0.97 - 1.53) | 0.12 | 65.9 | 0.03 |
| Feng et al. | Abortion number | CHD | 4 | 2,629 | | 1.31 (1.12 - 1.52) | (0.74-2.31) | 0.001 | 1.89*10^-8^ | 1.24 (1.09 - 1.41) | 0.07 | 54.9 | 0.45 |
| **Maternal parity** | | | | | | | | | | | | | |
| Feng et al. | Parity (as a binary variable) | CHD | 16 | 39,757 | | 1.01 (0.97 - 1.06) | (0.89-1.15) | 0.65 | 0.52 | 1.04 (1.01 - 1.08) | 0.02 | 53.4 | 0.3 |
| Feng et al. | Parity number | CHD | 14 | 38,027 | | 1.06 (1.02 - 1.09) | (0.96-1.16) | 0.001 | 1.80*10^-34^ | 1.09 (1.08 - 1.11) | 0.01 | 80.9 | 0.97 |
| **Paternal factors** | | | | | | | | | | | | | |
| Peng et al. | Paternal age<20 | CHD | 5 | 11,219 | | 1.24 (0.93 - 1.64) | (0.58-2.61) | 0.14 | 0.03 | 1.02 (0.78 - 1.32) | 0.13 | 38.9 | 0.81 |
| Peng et al. | Paternal age 20-24 | CHD | 5 | 11,219 | | 0.93 (0.78 - 1.11) | (0.50-1.72) | 0.4 | 7.39*10^-8^ | 0.82 (0.76 - 0.89) | 0.04 | 87 | 0.43 |
| Peng et al. | Paternal age 25-29 | CHD | 6 | 11,716 | | 0.89 (0.79 - 1.01) | (0.59-1.33) | 0.06 | 2.47*10^-5^ | 0.98 (0.94 -1.02 ) | 0.02 | 87.1 | 0.5 |
| Peng et al. | Paternal age 30-34 | CHD | 6 | 11,716 | | 0.98 (0.87 - 1.10) | (0.69-1.39) | 0.71 | 0.09 | 1.04 (0.97 - 1.12) | 0.04 | 76.6 | 0.14 |
| Peng et al. | Paternal age 35-39 | CHD | 5 | 11,219 | | 1.14 (1.06 - 1.22) | (0.98-1.33) | 2.45*10^-4^ | 8.53*10^-6^ | 1.13 (1.03 - 1.24) | 0.05 | 17.1 | 0.15 |
| Peng et al. | Paternal age >=40 | CHD | 5 | 11,219 | | 1.20 (1.05 - 1.38) | (0.76-1.90) | 0.01 | 2.06*10^-10^ | 1.27 (1.13 - 1.41) | 0.06 | 76.1 | 0.61 |
| Peng et al. | Paternal smoking | CHD | 10 | 10,588 | | 1.42 (1.17 - 1.74) | (0.71-2.84) | 4.57*10^-4^ | 9.46*10^-17^ | 1.01 (0.86 - 1.19) | 0.08 | 84.9 | 0.46 |
| Peng et al. | Paternal light smoking (1-9 cigarettes per day) | CHD | 4 | 3,772 | | 1.19 (0.82 - 1.71) | (0.24-5.98) | 0.36 | 0.01 | 1.58 (1.22 - 2.05) | 0.13 | 78.3 | 0.68 |
| Peng et al. | Paternal medium smoking (10-19 cigarettes per day) | CHD | 4 | 3,772 | | 1.41 (1.12 - 1.77) | (0.62-3.20) | 0.003 | 4.59*10^-5^ | 1.36 (1.04 - 1.77) | 0.14 | 42.8 | 0.78 |
| Peng et al. | Paternal heavy smoking (≥20 cigarettes per day) | CHD | 5 | 4,415 | | 1.76 (1.10 - 2.79) | (0.33-9.30) | 0.02 | 0.02 | 1.02 (0.86 - 1.20) | 0.09 | 85.6 | 0.02 |
| Peng et al. | Paternal wine drinking | CHD | 7 | 13,612 | | 1.48 (1.05 - 2.07) | (0.48-4.56) | 0.03 | 3.50*10^-8^ | 0.86 (0.69 - 1.07) | 0.11 | 87.9 | 0.54 |
| Zhang et al. | Paternal alcohol consumption | VSD | 6 | 11,775 | | 1.34 (0.99 - 1.83) | (0.52-3.47) | 0.06 | 0.45 | 1.07 (0.94 - 1.23) | 0.07 | 82 | 0.06 |
| Zhang et al. | Paternal alcohol consumption | ASD | 3 | 10,969 | | 2.60 (0.85 - 7.96) | (0.00-3257250.00) | 0.1 | 0.01 | 1.01 (0.83 - 1.23) | 0.1 | 93.5 | 0.07 |

^*^The effect sizes are expressed as relative risks (RRs).

^†^PI=prediction interval; p random=p value for random-effects meta-analysis; p fixed=p value for fixed-effects meta-analysis; ART, assisted reproductive technology; ASD, atrial septal defect; AVSD, atrioventricular septal defect; BMI, body mass index; CHD, congenital heart disease; COA, coarctation of the aorta; HLHS, hypoplastic left heart syndrome; IVF, in-vitro fertilization; PVS, pulmonary valve stenosis; SRI, serotonin reuptake inhibitor; SSRI, selective serotonin reuptake inhibitor; TGA, transposition of the great arteries; TOF, tetralogy of Fallot; TTTS, twin–twin transfusion syndrome; VSD, ventricular septal defect. .
